# Adaptation of Music Therapists’ Practice to the Outset of the COVID-19 Pandemic—Going Virtual: A Scoping Review

**DOI:** 10.3390/ijerph18105138

**Published:** 2021-05-12

**Authors:** Lucia Kantorová, Jiří Kantor, Barbora Hořejší, Avi Gilboa, Zuzana Svobodová, Matěj Lipský, Jana Marečková, Miloslav Klugar

**Affiliations:** 1 Center of Evidence-Based Education & Arts Therapies, Faculty of Education, Palacky University, Žižkovo nám. 5, 779 00 Olomouc, Czech Republic; lucia.kantorova@mail.muni.cz (L.K.); jiri.kantor@upol.cz (J.K.); zuzana.kelnarova@gmail.com (Z.S.); jana.mareckova@upol.cz (J.M.); 2 Institute of Special Education Studies, Faculty of Education, Žižkovo nám. 5, 779 00 Olomouc, Czech Republic; reditel@tloskov.cz; 3 Czech National Centre for Evidence-Based Healthcare and Knowledge Translation Institute of Biostatistics and Analyses, Faculty of Medicine, Masaryk University, Kamenice 753/5, 625 00 Brno, Czech Republic; klugar@med.muni.cz; 4 Centre for Social Services Tloskov, Tloskov 1, 257 56 Neveklov, Czech Republic; 5 Department of Music, The Faculty of Humanities, Bar-Ilan University, Ramat Gan 5290002, Israel; Avi.Gilboa@biu.ac.il

**Keywords:** music therapy, telemedicine, telehealth, remote therapy, COVID-19, adaptation, scoping review

## Abstract

*Background*: In the midst of a worldwide COVID-19 pandemic, music therapists previously not involved in telehealth had to develop effective remote forms of music therapy. The objective of this review was to systematically explore how music therapists previously working in-person adapted to the transfer to remote forms of therapy in the context of the coronavirus outbreak. *Methods*: We searched Scopus, Web of Science Core Collection, CINAHL, Medline, ProQuest Central, PubMed, EMBASE, PsycINFO and PsyARTICLES, grey literature (to October 2020), and websites of professional organizations. We followed the JBI methodology for scoping reviews. *Results*: Out of the 194 screened texts, we included ten very heterogeneous articles with an overall very low quality. Most texts described remote therapy in the form of synchronous video calls using the Internet, one paper described a concert in a patio of a residential home. We report the authors’ experience with the adaptation and activities, challenges and benefits of remote forms of therapy, recommendations of organizations, and examples and tips for online therapies. *Conclusions*: Music therapists have adapted the musical instruments, the hours, the technology used, the therapeutic goals, the way they prepared their clients for sessions, and other aspects. They needed to be more flexible, consult with colleagues more often, and mind the client-therapist relationship’s boundaries. It seems, when taken as a necessary short-term measure, online music therapy works sufficiently well. The majority of papers stated that benefits outweighed the challenges, although many benefits were directly linked with the pandemic context.

## 1. Introduction

The World Health Organization declared the spread of the new SARS-CoV-2 virus as a pandemic on 11 March 2020 [1], at a time when the Western countries were one by one going into “lock-downs”. Services that were not deemed essential were closing, and people were advised to stay at home. Where possible, work was shifted into home offices and virtual platforms. According to an online survey, for more than half of US-based music therapist survey respondents (about 54.4%), this mainly meant providing clinical services from home [2], despite frequently having no prior experience or knowledge of telemedicine. Furthermore, (a theme that lies outside of the scope of this paper) some music therapists were able to keep working without changes in-person, having to deal with a specific set of issues related to preventive measures against the spread of COVID-19, some were given different duties on-site, while some were laid off or their positions were canceled completely. [2] Adaptation of music therapy (MT) to remote sessions has proved a multifaceted challenge, and many professional organizations quickly addressed the issue. [3] To state only a couple of examples, the American Music Therapy Association (AMTA) established a COVID-19 task force early on and has since been providing support and encouragement, as well as practical tips for music therapists [4]. Meanwhile, the British Association for Music Therapy (BAMT) was also swift to respond to the situation and has a plenitude of well-structured resources and advice on their website [5].

The pandemic has highlighted the role of virtual music therapy (VMT) and digital telehealth tools [6], which had been used even before the pandemic [7]. VMT had, in the pre-COVID era, been utilized for specific reasons in some populations: veterans, those with mobility issues or living in remote and rural areas, to name a few, but had never been applied widely, without having many alternatives and without much preparation. Since the strike of the pandemic, the remote forms of music therapy have made it possible to continue addressing the needs of the broad spectrum of clients. Many music therapists have been forced to use digital tools [3]. In addition to the already enrolled clients, the pandemic has been associated with the need to address additional groups of clients with pandemic-associated exacerbations of their preexisting conditions, new-onset conditions related to isolation and instability, and stress-related conditions experienced by those in the forefronts of the pandemic battle [8,9].

What was needed was a vast and immediate transfer of music therapy services into a virtual setting, often without the necessary equipment and environment, without the desired experience on the part of both the therapists and the clients, and even for client groups that were not among those that could easily be considered suitable for virtual therapies. Therefore, we felt exploration of the insights gained by music therapists in this process would help at this time. Now, over some nine months into the pandemic, we have attempted to prepare an overview of the accumulated experience. We thought music therapists and researchers would have had enough time to try various remote therapy techniques, reflect on them, and publish their experiences and insights. We suspected there might not be an abundant number of papers published at this time, although we knew some existed based on a preliminary search. Therefore, we decided (a) to conduct a comprehensive and robust database and manual search and to include all relevant texts disregarding their design or quality, (b) to complement the search results by an overview of advice given by music therapy associations, and (c) to prepare this paper as quickly as possible, without sacrificing its methodological rigor, so its results could be up-to-date at the time of publishing. We intend to conduct an update in a year and one reviewer (LK) is appointed to initiate the process.

For this purpose, a scoping review was considered the most appropriate method. It was conducted according to the JBI methodology [10,11] and in line with the PRISMA recommendations [12,13]. The objective of this paper is to explore, in a broad way, the experiences that have accumulated during the COVID-19 pandemic in adapting music therapy services, as well as to provide an overview of recommendations and advice given by music therapy associations.

### 1.1. Review Question

Review question in PCC format (established and published a priori): How did music therapists previously working in-person (Population) adapt to remote forms of music therapy (Concept) in the context of the COVID-19 pandemic (Context)? (What advice did music therapy associations provide in this situation)?

### 1.2. Eligibility Criteria

#### 1.2.1. Participants

Music therapists previously working in-person with their clients. The term music therapist is defined very broadly for the purpose of this review. There is no limit on the type of clients as we did not expect to find many relevant texts in the still early stages of the pandemic and the aim of this scoping review is to provide a comprehensive map of the researched topic.

#### 1.2.2. Concept

Adaptation to a remote (not in-person, not face-to-face) form of music therapy—the experience, the methods, techniques used, the necessary equipment, the costs or barriers, and guidance of professional organizations.

#### 1.2.3. Context

The COVID-19 pandemic with a long-lasting or recurrent lock-down and the need to transfer therapeutic services to a virtual form; there is no limit on the setting; this review does not focus on music therapeutic methods/services that were remote before the pandemic; it also does not cover specifically the experience of clients with music therapy or the use of music therapy to relieve stress caused by the pandemic; it does not include experience with an in-person form of therapy using protective equipment against any COVID-19 infection.

#### 1.2.4. Types of Sources

Any texts, including quantitative, qualitative, mixed methods, both published and unpublished, grey literature, with abstract in English, limited to the publication year 2020, no geographical limit.

#### 1.2.5. Databases

Scopus, Web of Science Core Collection, CINAHL, Medline, ProQuest Central, PubMed, EMBASE and PsycINFO with PsyARTICLES, Google Scholar and other grey literature, the official websites of North American, Australian, New Zealand, European, Japanese, Israeli, and international music therapy professional organizations. A comprehensive manual search of music therapy journals and blogs.

## 2. Materials and Methods

### 2.1. Study Design

We drafted the paper using the methodology described in the Joanna Briggs Institute Reviewer Manual [11]. We followed the PRISMA 2020 statement: an updated guideline for reporting systematic reviews [12,13]. (Supplementary S1) We drafted the protocol prospectively and, after finalization, registered with the Open Science Framework on 15 November 2020 (https://osf.io/y948u/https://osf.io/kv9hu/, accessed on 25 December 2020) [14]. Epistemonikos was searched to find any scoping or systematic reviews or protocols on the delivery of MT during the pandemic with no results.

### 2.2. Search Strategy

We conducted a comprehensive search of both published and unpublished literature in the following databases with a limit to the year 2020, on 25 October 2020: Scopus, Web of Science Core Collection, CINAHL, Medline, ProQuest Central, PubMed, EMBASE, and PsycINFO with PsyARTICLES; and in grey literature databases: the Cochrane Library (The Cochrane Central Register of Controlled Trials), DART Europe E-theses Portal, Open Access Theses and Dissertations, Open Grey, Electronic Theses Online Service, Clinical trials & Current Controlled trials, and Google Scholar. The search strategies were drafted by a researcher and a librarian [LK and ZS] and further refined through team discussion. The final search results were exported into EndNote X9.2 (Clarivate Analytics, Philadelphia, PA, USA), and duplicates were removed. The electronic database search was supplemented by searching the leading music therapy journals and a manual search of music therapy organizations’ websites and reference lists of included studies by two independent researchers [LK and BH], finalized by the end of November 2020. The search was not limited by language nor by geographical location. Databases were searched only for the year 2020, to eliminate texts on virtual music therapy not related to the pandemic. The final search strategy for all databases and a list of websites, journals, and other searched sources can be found in Supplementary S2.

### 2.3. Eligibility Criteria

A piloting phase of screening and data charting was performed to increase consistency. The two reviewers responsible for independently screening texts and charting data [LK and ZS] met weekly to compare results, comment on progress and resolve any difficulties. As needed, a third reviewer [JK] was called to settle any disagreements. During the screening of the full texts, we excluded telehealth articles unrelated to COVID-19 or music therapy. We did not include texts dealing only with technology, laws, or standards of practice. We included texts on the adaptation of music therapists during the pandemic in various settings: educational, freelance, or clinical. We included texts on the education of music therapy students only when therapy sessions were conducted and described. We did not address the issue of continuing in-person music therapy during the pandemic. We included texts even if only part of the content was found relevant. We have decided to include papers that could be considered “near-miss”, e.g., a paper on delivering therapy via a concert performed in a residential home patio (with social distancing, without the need to use protective equipment).

### 2.4. Data-Charting and Presentation

Data-charting also underwent a piloting phase, during which we refined the data-charting tool developed and published in the review protocol. It became more detailed as we saw what kind of information the authors of texts were reporting.

We independently abstracted data on author, month and year of publishing, language, country of origin and setting, type of text and database, the methodology of the paper, the target client group of the provided services, the population that was researched (focused on) in the paper (including age, gender, profession, sample size, and general characteristics), any specific music therapy techniques used, any specific examples of working with the clients, main and additional objectives, primary and additional outcomes/results (those relevant to the review question), recommendations for music therapy practice and other recommendations, and conclusions. We felt the data-charting phase should be thorough and detailed as the included texts were quite heterogeneous as to their type, study design, methodology, and sample. We also charted recommendations from the websites of professional organizations. It should be stated here that we identified more relevant content, however, we have decided to extract only data with unlimited access. In other words, we have not charted data from web content available only to registered members of associations. The two reviewers discussed the compiled data and prepared an outline of data presentation tables (Supplementary S3 and S4). A draft of the paper was then prepared by LK and discussed with the whole team. Adjustments were made accordingly, and the final version was completed. Below are the main findings of the scoping review.

## 3. Results

After duplicates were removed, a total of 194 citations were identified from searches of electronic databases and other sources (journals, websites, reference lists). Based on the title and the abstract, 177 were excluded, with 17 full-text articles retrieved and assessed for eligibility. Of these, seven were excluded for the reasons stated in the PRISMA flow chart (Supplementary S1). We also searched the list of 543 included articles in a recent scoping review on telehealth services provided during the pandemic and found 0 relevant results concerning music therapy [15]. The results are presented below separately for (a) outcomes related to the review questions (all sources) and (b) recommendations and advice (only from official websites of significant MT associations) (Supplementary S4).

Ten articles were included in this scoping review, eight of them identified through database search (published and unpublished sources) [2,16,17,18,19,20,21,22] and two identified through a manual search of websites of MT associations [23,24]. The basic characteristics are in Table 1 and all data-charting tables and a data summary table are in Supplementary S3. Below is a narrative and tabular description of the scoping review results concerning the adaptation of MT to the pandemic.

Most texts, all English, were from the USA, and the rest were from Europe. Some were newspaper articles or flyers. The settings ranged from educational settings (primary, secondary, and university) to healthcare (outpatient clinic, hospitals) to social services (retirement homes). A similarly wide range was observed for client groups. Most texts did not provide information on specific clients and sessions held during the pandemic, and instead, they often described their COVID-related adaptations in a general way. The client groups identified in the relevant texts were: Seniors and aging adults (two texts); Autism Spectrum Disorder (two); Developmental disabilities and challenges (two); Neurological diseases and injuries (two); Healthy university students (one); Psychiatric disorders, interpersonal dysfunctions and problems, sleep disorder (one); Unspecified (three); Infants at Intensive Care Nursery and their parents and relatives (one). The spectrum of the study designs and texts was also broad, ranging from newspaper articles and flyers, to surveys and models. The papers were quite heterogeneous in their design, methods, client groups, or setting. The outcomes did not contain contradictory statements. Most texts described remote therapy in the form of synchronous video calls using the Internet, while one paper performed a concert in a patio of a residential home.

We intended to chart information on music therapists’ experience, therapy techniques, equipment needed for the therapy, the costs and barriers, and guidance from professional organizations related to the adaptation of services during the pandemic (remotely). Some of these expected outcomes were not widely reported, such as equipment/technology and costs and barriers, at least not in a concrete way, while other outcomes were reported often, such as experience, opinions, challenges, recommendations, and guidance. We provide the results below in a tabular form: describing the various adaptations of services, benefits, and challenges reported by therapists, and the recommendations extracted from websites of professional organizations.

All ten texts provided information on the experience of music therapists who needed to adapt their services to remote forms of therapy due to the COVID-19 pandemic (see Table 2 below). They had to adapt their therapeutic goals, the way they prepare/brief a client before sessions, find new forms of delivering therapy, adapt their home lives and settings, and be more flexible in handling the hours, the clients, and the activities used during the sessions. Many used improvisations during video calls with ordinary household items to create music. There was much learning, an increased need to consult with colleagues, the need to deal with new questions, such as privacy, security, and availability of the technology. An issue of work-life balance and maintaining boundaries was raised as well. The findings can be found in Table 2 and the full data charting tables and recommendations in Supplementary S3 and S4.

We identified statements on benefits and challenges of remote forms of therapies (in these cases, online synchronous video calls), as perceived by music therapists, in eight papers. In a tabular form, below are the results related to the identified benefits and challenges divided into a few sections for clarity (Table 3).

We performed a manual search of major MT associations’ websites (a list is provided in Supplementary S2 and results in Supplementary S4) and identified several webpages from three associations providing advice and recommendations to music therapists. We compiled a list of resources for MT practices and techniques that can be used during remote sessions, which is available in Supplementary S5 in the form of a short description of the content and the source URL.

## 4. Discussion

### 4.1. The Overall Quality of the Included Texts

Of the ten included texts, some were newspaper articles; one was a flyer. There were opinions/essays, case reports, and surveys, most not of high methodological quality. We did not formally appraise the overall quality of the texts, but it can be estimated to be very low. Some aspects of the review question were not addressed directly in the included texts, such as costs, barriers, and equipment needed, but could be assumed from the evidence. We aimed to identify what was the experience of those music therapists who had to adapt their practice to remote forms due to the pandemic. There was great heterogeneity in how this question was answered in the texts, although we would like to emphasize, the experiences and opinions of the therapists were not contradictory, which adds reliability to the findings. Overall, a lot can be learned from the scarce evidence at this, still not final, stage of the pandemic.

### 4.2. The Music Therapists’ Experience with Adaptation to Remote Therapy

From the findings, we can see that a lot of the positive attitude for the change of services from in-person to remote form came from the gratitude of music therapists to be able to continue providing services during the pandemic. This was reflected in the change of the therapeutic goals: to bring joy, to focus on what can be done, and to relieve stress. Both therapists and their clients had to adapt their surroundings, home life, and improve their skills using technology. They had to be more mindful of where the sessions are being held, if the lighting, microphone, and audio are set up well, what is in their background and if they are in a quiet space with no interruptions. Therapists needed to learn to work with new aspects of technology and had to be flexible to enable a variety of client groups to join. Not only the novelty of the experience but also the specifics of this form of therapy could be challenging, such as trouble with the internet connection, breaks in the line, weird sounds, asynchronized harmony and rhythm, talking over each other, and disconnections of the call. The issue of boundaries was raised, both in the more negative sense that the therapist was at risk of losing boundaries in the client-therapist relationship and in a positive sense, the therapist could see more of the client’s life and that the barriers between therapy and real-life became thinner.

There was a multitude of techniques and practices mentioned, ranging from asynchronous remote therapy in the form of prerecorded videos and playlists, to texting the therapist, to using the hospital bedside phone, to synchronous video calling using laptop and the Internet, providing concerts, doing finger play songs and age-appropriate songs, incorporating sign language and dancing, and many others. The articles we found reflected experiences and music therapy techniques for both individual and group therapy. There are more challenges with music therapy groups. Some of the strategies used may include turn taking/musical dialogs, simultaneously working with non-rhythmical structures or muting participants. According to the experience of authors, music therapy online may be more structured, require more time, and may be less spontaneous than face-to-face therapy.

Many papers mentioned using home-made musical instruments, ordinary household items, or available surfaces or the body for drumming. Of course, limitation concerning musical resources and sharing high quality instruments is a crucial problem for many practicing music therapists, because it might affect the aesthetic and healing quality of sound/music and attraction of music to clients, especially for music therapists or clients who are sensitive to sound quality.

Another more frequent statement was that it was not necessary to continue sessions exactly as before, and using no music was also acceptable; clients could prefer singing, or just talk. It should be noted, though, that in most music therapy approaches, rarely is music neglected altogether. In cases where therapy is verbal only, we may question whether this is music therapy or a different mode of therapy. Therefore, music therapy practitioners should be encouraged not to cease involvement of therapeutic musical experience into the virtual music therapy sessions and to learn how to properly implement music in an online environment. Papers emphasized that therapists needed to be flexible, both in the sense of the technology used, and the therapeutic activities and the form of therapy. Another requirement for the music therapists was related to the logistics. The therapists needed to hold frequent consultations with colleagues, to plan the sessions ahead and be flexible, to deal with the problems with the Internet and technology, to maintain work-life balance and boundaries, and to make changes in scheduling of sessions considering a possible “Zoom fatigue”.

### 4.3. The Benefits and Challenges of Remote Therapy

Three papers reported that the benefits of having remote online sessions outweigh the challenges encountered. [2,18,19] The rest of the papers did not provide this information, and none stated the contrary. In this regard, it should be noted that two papers [2,19] thought that VMT should be viewed as an alternative and not a substitute for in-person therapy, an opinion in line with the guidance provided by MT associations. Roughly about twice as many benefits were mentioned than challenges, although this might reflect the fact that some music therapists did not have access to online sessions [2], and experience could still be scarce. Moreover, about half of the identified benefits were directly related to the pandemic context and would not be relevant for providing virtual sessions under other circumstances. Patients’ regard for such a form of therapy and their engagement have increased out of gratitude for the possibility to continue therapy in any form–a scenario that is also unlikely to continue when in-person therapy is again possible. The same applies to therapists who were grateful for the opportunity to continue earning money in the crisis. Nevertheless, there are still some obvious benefits, such as enabling access to therapy for specific groups of clients, the possibility to see more of the patient’s life, the benefits for hard-to-move patients in institutions and hospitals and group therapy, and the possibility to engage more family members and interpreting services. This reflects the findings on virtual MT before the pandemic. [25,26] Furthermore, these benefits are only heightened under crises such as was brought on by the COVID-19 pandemic.

Some challenges mentioned in the papers were related to the novelty of the experience and could be overcome if virtual forms of therapy were used more. Among these were: online therapy may feel weird and isolating for the client, Internet’s lag-time disrupts the synchronization and rhythm, technology can be hard to learn; some companies do not allow telehealth options. Other challenges would be harder to overcome: virtual therapies are not appropriate for all clients (e.g., children require to set a time limit for screen time), can be cost-prohibitive, are connected with barriers and inequities, and there is a risk of losing boundaries in the client-therapist relationship. Moreover, we would like to increase awareness about some differences between face-to-face and virtual music therapy. A virtual setting challenges some important principles of music therapy—synchronization in musical contact, working with physical proximity and distance (e.g., in children with autism), usage of tactile qualities of instruments, deteriorated quality of sound and its timbre, lack of resonance, etc. For these reasons, music as a therapeutic agent doesn’t work in the same way, comparing online and physical setting. Music therapy as a relation therapy may lose important aspects based on physical contact when transferred to the virtual space.

These may be some of the reasons that many practitioners prefer to decline their practice altogether than to provide therapy with a level of quality that might be considered as superficial and deteriorated. However, we are convinced VMT is needed for many clients during the pandemic, especially if it is meant as a temporary option that is preferrable to abandoning the practice.

### 4.4. The Advice of the MT Associations

The advice and recommendations of the three MT associations that provided and made freely available this kind of information were very similar to each other. There was only one contradictory recommendation identified. The methodology of developing recommendations was not reported. We found some contradictions between the evidence accumulated in this scoping review and some of the identified recommendations, e.g., that remote sessions should take place at the same time (Supplementary S4) vs. that therapists needed to be flexible and adjust their hours and length to the clients’ needs and consider possible Zoom fatigue (Table 1).

We think an obvious conclusion of our findings is that tele-music therapy should continue to be used in the field and developed even more. However, we need to keep in mind the challenges, some of which are unlikely to be overcome, associated with remote forms of therapy, and the fact that many of the currently perceived benefits of teleservices are directly linked with the pandemic situation. However, because the adverse effects of VMT are yet under-researched, practitioners should be cautious about implementing these new methods. As noted before, some very basic elements of music therapy might be hindered with VMT (e.g., the ability to play together, to synchronize, quality of sound, quality of interpersonal connection), and until the effect of this is systematically researched, VMT should be applied to non-pandemic contexts only with great caution.

### 4.5. Limitations and Strengths

The authors of this scoping review comprise a wide team of experts in various related fields–a medical doctor, an epidemiologist, methodologists (for systematic reviews and clinical practice guidelines), music therapists, music therapy educators, special educators, a psychotherapist, and a physiotherapist. Although no expert on telehealth was involved most of the authors have practical experience with virtual music therapy during the pandemic:

AG was a lecturer in an online music therapy training “Musical Dialogue in the time of Corona” (with Elana Baruch).

BH has personal experience as a student of a music therapy program that has been delivered online since the start of the pandemic.

JK is an author of an evidence implementation project focused on the adaptation of music therapy practitioners in the Czech Republic to the online setting. He was also responsible for adaptation of a MT university program (at Palacky University Olomouc) to the online setting.

ML adapted his community music therapy practice (involving radio and community concerts) to the online setting in the residence facility for adults with intellectual disabilities (Center Tloskov).

This scoping review, although aiming at an understudied (or under-published) area, having included only ten heterogeneous text, of which some were newspaper articles or flyers, was conducted very quickly and could be considered a rapid scoping review. However, due to their speedy development, rapid reviews usually sacrifice some of the rigorous methodology, which was not the case here. This scoping review was based on a robust and systematic search of a wide range of literature, both published and unpublished sources, complemented by an extensive manual search and a compilation of recommendations of MT associations and resources for music therapists. We have also aimed at achieving a high level of transparency, and all our data are provided in Supplementary Materials. Since the search, more texts were published, and these will be incorporated in an updated review within a year.

### 4.6. Implications for Future Research

In this review we were looking for the immediate reaction of music therapists to the COVID-19 crisis. These reactions are assumably more intuitive and spontaneous than reactions that come later, after things have settled and/or new frameworks of treatment substitute the old ones. We were looking for the raw reactions of our peers around the world as conditions were rapidly changing and flexibility, creativity, and innovation were called upon. This is why we limited the literature review survey to a short period of several months after COVID-19 began. In future studies we believe it is important to focus on later articles that referred to this topic and to compare them with the earlier articles that we surveyed here.

Although there is evidence of the effectiveness of applying digital tools in MT for some client groups, more research is welcome that would be specific to the pandemic and the client populations that have not been explored yet. [25,27,28,29] We encourage music therapists and related institutions/organizations to review their experience with adapting their services to the pandemic and to publish their findings, insights, models, suggestions, and recommendations, providing practical tips and descriptions of techniques and technology used, together with important background information, such as their previous services, their target client group, and their setting. More evidence is needed regarding the costs and barriers, and risks.

Another question that needs to be addressed is how effective remote music therapy is, compared to in-person delivery for the various client groups in the context of the pandemic. Both delivery and recipient perspectives need to be assessed in research when analyzing the similarities and differences between face-to-face and remote sessions. Moreover, we have encountered limited reporting of risks.

Researchers and associations have already begun addressing the issue of adapting to the post-pandemic era, both in the sense of resuming in-person MT and a broader perspective of all the implications on MT. Models for restoring practice need to emerge for various settings, where MT is used (clinical, educational, hospice), and specific and newly encountered issues, as they arise, should be addressed, e.g., problems related to the Covid-19 pandemic [9,30].

Recommendations for telehealth in music therapy should be based on evidence, whether scientific or expert [31] or a combination, and could be inspired by existing recommendations on telehealth [32,33]. The GRADE system [34] for developing recommendations can be used in the music therapy field.

We recommend to also explore other possibilities of remote music therapy that are not covered in this review, e.g., providing information sources with music for improving the wellbeing during the pandemic or for improving the self-care of parents and caregivers [23]. In extreme lockdown conditions with no steady internet communication, such possibilities could be the only way to get to and help clients, and it is important to know whether there is good experience with such techniques or whether they are problematic.

For the development of music therapy theory in the more distant future, it will be necessary to explore thoroughly the differences between virtual and face-to-face music therapy. We should be aware that virtual music therapy challenges foundational postulates of music therapy and there are noticeable differences between those forms of delivery. Furthermore, we shouldn´t label as VMT the sessions without any musical intervention.

The conclusions of this review may be relevant also for telehealth practices of a wide range of professionals.

## 5. Conclusions

In this scoping review, we identified and included ten very heterogeneous texts on the adaptation of music therapy services during the COVID-19 pandemic. Furthermore, we compiled the recommendations of three music therapy associations that had advice freely available on their websites. We encountered some contradicting advice with the evidence brought by this review. The music therapists adapted to remote services quite well, with the majority stating that benefits outweighed the challenges. However, many benefits were directly linked with the pandemic context and would unlikely be applicable when the crisis was over. They have used various forms and activities during the sessions, adapting the musical instruments, the hours, the technology used, the therapeutic goals, the way they prepared their clients for sessions, and other aspects of the therapy. They needed to be more flexible, consult with colleagues more, and mind the client-therapist relationship’s boundaries. We hope more music therapists will publish their experience. More research is needed on the costs, barriers, inequities, and risks of remote forms of therapy. Recommendations should be based on scientific or expert evidence and transparently reported, preferably using the GRADE system. This review’s conclusions may not be relevant only for music therapists and associations but also for telehealth practices of a wide range of professionals.

## Figures and Tables

**Table 1:** Characteristics and summary of included texts.

Characteristics and Summary of Included Texts	
Author, Date, Country, Design, Type of Text	Setting and Client Group
Gaddy [2]	N = 1196 (music therapists)
COVID-19 and Music Therapists’ Employment, Service Delivery, Perceived Stress, and Hope: A Descriptive Study	Private Practice/Contractual: 37.38%; Schools: 24.80%; Hospice: 19.43%; Psychiatry: 19.00%
September 2020, USA	
Cross sectional study (survey of music therapy professionals), Journal article	
	Autism Spectrum Disorder: 44.73%; Developmental disabilities: 44.02%; Older Adults: 35.85%; Alzheimer’s: 34.35%
Sasangohar [16]	An outpatient psychiatric clinic
Adapting an Outpatient Psychiatric Clinic to Telehealth During the COVID-19 Pandemic: A	Patients with psychiatric disorders, interpersonal dysfunctions and problems, sleep disorders
Practice Perspective	
October 2020	
Case study, Journal article	
Knott [17]	Healthcare systems,
Virtual Music Therapy: Developing New	Educational setting and communities
Approaches to Service Delivery	Any client of music therapy
September 2020, USA	
Journal article	
The Enclave at Round Rock Senior Living Hosts a Patio Music Party with the Help of North Austin Music Therapy [18]	Retirement home
March 2020, USA	Seniors, aging adults, ASD, developmental challenges and neurological diseases and injuries
Newspaper article	
College of Music to Continue Successful Virtual Tele-therapy Services [19]	Educational setting
August 2020, USA	Students and young adults with disabilities
Case report, Newspaper article	
Negrete [20]	Hospital environment
Meeting the Challenges of the COVID-19	Infants at Intensive Care Nursery and their parents/relatives
Pandemic: Virtual Developmental Music	
Therapy Class for Infants in the Neonatal	
Intensive Care Unit	
July–August 2020, USA	
Case report, Journal article	
Music School’s Virtual Approach Proves a Hit [21]	Educational setting
June 2020	
Newspaper article	Regular students and students who need additional support
Gupta [22]	Educational setting
Singing Away the Social Distancing Blues: Art Therapy in a Time of Coronavirus	University (undergraduate art therapy program)
September 2020	
Case report, Essay by a psychology professor	
Berman, EMTC [23]	Music therapy association
Music Connects Us	Music therapists and music therapy clients
September 2020	
Flyer	
North London music therapy [24]	Music therapy session within North London music therapy organisation
Music Therapy in the Time of COVID—How NLMT’s Remote Sessions Actually Work	
Website article	Music therapists and their clients in online therapy

**Table 2:** Summary of adaptations of music therapists to the COVID-19 pandemic.

Summary of Adaptations of Music Therapists to COVID-19 Pandemic	
**Adaptation of the goals:**	
	Additional goals also aimed at team members and community needs: during the pandemic people needed to be together and share. [18]
	To focus on what they can do and not what they can’t do, on feelings of happiness, social, emotional, and physical connection through music. [18]
	Promote accessibility for everyone at home to make music, have fun, and still be creative. [21]
	To show how music relieves stress and brings joy and harmony during the pandemic, it reminds people they are not alone. [22]
	Use music to structure the day, reduce social isolation, stress and bring more joy during lockdowns. [23]
**Preparing the client:**	
	You [client] have to rely on a good internet connection; sometimes there are breaks in the line or weird sounds; sometimes you can’t quite hear what your therapist said or played; sometimes your therapist can’t quite hear you; sometimes the call gets disconnected. [17]
	You [client] need to consider the room you’re in quite carefully—quiet, so not much background noise, and that’s unlikely to be disturbed for the duration of your session; background, good light. [17,24]
**Adaptation concerning the space:**	
	Sessions at home. Try to find a working space at home, which is only used for work. [16]
	Sessions outside–residents of a senior home were on their balconies and therapists on a patio (enabling social distancing). [18]
**Methods and techniques:**	
	Making weekly 10-min videos for children and posting them on YouTube. Incorporating MAKATON into music videos. [21]
	Remote sessions over the phone or online, using secure software programs. [23]
	Respondents reporting changes in employment (n = 886) were most often providing remote clinical services from home (67.40%). [2]
	An interactive concert for residents of a retirement home—session on balcony and patio of a residential home. [18]
	Using household items and DIY instruments. [19,21,22,23,24]
	Active music-making (solo improvisation [22,24], musical dialogues, a verbal reflection of music made) [19], movement to music, songwriting and composing (e.g., adding lyrics to instrumental music or loops [23]) and song discussion, listening to music (e.g., a music-assisted relaxation [17]) and creation of audio/video recordings with clients [17].
	Reflecting verbally on the music made and feelings that arise during the crisis. [23]
	New adaptations of methods and techniques to fit the pandemic situation, e.g., playlists for the structure of the day [23], sending a song to family and friends to stay connected, or selecting songs through touching the screen of the computer.
	Improvisational virtual jam session. [22,23,24]
	Adaptations connected to the usage of technologies (e.g., selection of songs through touching the screen of the computer. [17]
	Texting. [16]
	Among those who reported delivering alternative services compared to pre-pandemic: Telehealth services (54.81%), virtual music lessons (17.01%), prerecorded songs/playlists (16.98%), and prerecorded video sessions (16.00%). [2]
	Identifying preexisting content (audio, videos, and music-making instructions) readily available online. [17]
	Fingerplay songs, Preschool and early childhood music; Relaxation-oriented audio and video recordings; Utilizing an adult patient’s bedside phone, a music-assisted relaxation intervention; guided imagery, breathing techniques, and mindfulness-based strategies. [17]
	One text reported that no adaptation of MT techniques was needed (Intensive Care Nursery for infants/their parents). Songs that introduced socialization. Used singing and shakers. Songs taught baby sign language. Before each song, the music therapist would educate the parents on how to participate in the song with their child, providing hand-over-hand support, pointing to the different body parts the song was cueing, explaining how the song supported their infant’s development and demonstrating how the song could be used when not in a group setting. [20]
**Adaptation of musical instruments:**	
	Using ordinary household items to make music. [18,21,22]
	Making a noisemaker from an Easter egg. [18]
**Technologies:**	
	Leverage multiple platforms and modalities to facilitate the initial transition, Zoom, Skype, Webex, FaceTime, occasionally texting with the therapist. [16]
	It is useful to wear headphones or even an audio interface for each telepractice. [16,17,24]
	Recording sessions in case the session is interrupted—especially in group therapies. [16]
	Don’t use a personal account. [16]
	Identify at least one on-site staff member who can help families troubleshoot technical difficulties. [20]
	A laptop is preferable to a phone. [17]
	Creation of a new model for implementing the telepractice (the Three-Tiered Scaffold Model). [17]
**Flexibility and logistics:**	
	Adopt a suitable system of work to different clients—choice of the communication channel, hours, length, etc. [16]
	Future music therapists need to be flexible. [19]
	Interruptions may disrupt session plans and require management. [16]
	Necessary to incorporate additional consultation meetings between colleagues (therapists). [16]
	Maintain work-life balance by separation of space and time. [16]
	Acknowledge that there is such a thing as Zoom fatigue. Therefore, it is important to plan out the hours spent online wisely. [16]
	The online class also minimizes the amount of staff time required to help with the class because all the family or nurse needs to do is to log on. [20]

**Table 3:** Summary of benefits and challenges when adapting to remote sessions, as reported by music therapists.

Summary of Benefits and Challenges when Adapting to Remote Sessions, as Reported by Music Therapists	
	It is an alternative, rather than a substitute for in-person therapy. [2,19,23]
	Benefits outweigh the challenges. [2,18,19]
**Benefits (not necessarily associated with the pandemic):**	
	Benefits for the training of music therapists (teaches them to be flexible). [19]
	Easy access for clients [19]; e.g., remote locations or immunocompromised [2]; for clients in the hospital setting on respiratory support with difficult transportation, e.g., for the infants with tracheostomies, or those on isolation. [20]
	The expansion of boundaries, which provides a directly contextualized view into patients’ home lives. [16]
	Using technology spurs creativity–a lot can be done. [2]
	The amount of staff required to assist with organizing MT in a hospital setting decreased. [20]
	Off-site family members of hospitalized children in therapy were able to attend. [20]
	Interpreting services could connect easily. [20]
**Benefits directly related to the COVID-19 pandemic:**	
	It brings joy and love to overpower the terror of COVID-19. [22]
	Benefits related to the possibility of avoiding the protective measures: Masks and social distancing disrupt both verbal and non-verbal communication, can muffle voices, and limit a therapist’s assessment of patient affect. [16,20]
	Engagement in some respects has increased, virtual groups have been well attended—due to the isolation caused by the pandemic, patients were enthusiastic about finding different channels of communication/provided therapy. [16]
	Opportunity to continue earning money and providing services. [2]
**Challenges:**	
	It feels weird [for the client] at first. [17]
	It can feel very isolating [for the client]. [17]
	Problems with the Internet and technology–Internet’s lag-time causes delays in rhythm and harmony not in perfect synchronization. [22,23]
	Risk of losing boundaries in the client-therapist relationship due to the home environment. [16]
	Virtual service technology is difficult to learn. [2]
	Virtual service technology is not appropriate for all clients. [2]
	Virtual service technology is cost-prohibitive for many clients and clinicians. [2]
	Connected with barriers and inequities, which keep clients from accessing these services. [2]
	Not all companies/organizations allow telehealth options. [2]
	For child clients, there is a need to set a time limit for screen time. The therapist had to look for signs of overstimulation. [20]
	It may not be possible to offer music therapy exactly as usual. [23]
**Clients’ feedback:**	
	Pleased to have the option of continuing therapy in some form. [16,17]

## Data Availability

All data is in Supplementary Materials.

## Supplementary Materials

The following are available online at https://www.mdpi.com/article/10.3390/ijerph18105138/s1, Supplementary S1 PRISMA Checklist 2020, Supplementary S2 Search strategies, sources and results, Supplementary S3 Data charting tables for individual papers and a summary of data table. Supplementary S4 Data charting table from websites and Recommendations, Supplementary S5 Tips for music therapy activities.

## References

[1] World Health Organization (2020). WHO Director-General’s Opening Remarks at the Media Briefing on COVID-19-11 March 2020.

[2] Gaddy S., Gallardo R., McCluskey S., Moore L., Peuser A., Rotert R., Stypulkoski C., LaGasse A.B. (2020). COVID-19 and Music Therapists’ Employment, Service Delivery, Perceived Stress, and Hope: A Descriptive Study. Music Ther. Perspect..

[3] Carvajal M.A. (2020). Telehealth Music Therapy: Considerations and Changes During the Covid-19 Crisis.

[4] AMTA COVID-19 Resources for Music Therapists and Students. https://www.musictherapy.org/about/covid19_resources.

[5] Therapy B.A.F.M. Covid-19—Directory Including BAMT Guidance. https://www.bamt.org/about-british-association-for-music-therapy/covid-19-useful-information.html.

[6] Ohannessian R., Duong T.A., Odone A. (2020). Global Telemedicine Implementation and Integration within Health Systems to Fight the COVID-19 Pandemic: A Call to Action. JMIR Public Health Surveill..

[7] Glover K.K. (2020). A Phenomenological Study of the Therapeutic Relationship in Tele-Music Therapy in the US. https://digitalcommons.molloy.edu/etd/85/.

[8] Chen J.A., Chung W.-J., Young S.K., Tuttle M.C., Collins M.B., Darghouth S.L., Longley R., Levy R., Razafsha M., Kerner J.C. (2020). COVID-19 and telepsychiatry: Early outpatient experiences and implications for the future. Gen. Hosp. Psychiatry.

[9] Mastnak W. (2020). Psychopathological problems related to the COVID-19 pandemic and possible prevention with music therapy. Acta Paediatr..

[10] Peters M.D., Marnie C., Tricco A.C., Pollock D., Munn Z., Alexander L., McInerney P., Godfrey C.M., Khalil H. (2020). Updated methodological guidance for the conduct of scoping reviews. JBI Evid. Synth..

[11] Peters M., Godfrey C., McInerney P., Soares C., Khalil H., Parker D. (2015). The Joanna Briggs Institute Reviewers’ Manual 2015: Methodology for JBI Scoping Reviews.

[12] Page M.J., Moher D., Bossuyt P., Boutron I., Hoffmann T., Mulrow C., Shamseer L., Tetzlaff J., Akl E., Brennan S.E. (2021). PRISMA 2020 Explanation and Elaboration: Updated Guidance and Exemplars for Reporting Systematic Reviews. BMJ.

[13] Page M.J., Moher D., Bossuyt P., Boutron I., Hoffmann T., Mulrow C., Shamseer L., Tetzlaff J., Akl E., Brennan S.E. (2021). Updating guidance for reporting systematic reviews: Development of the PRISMA 2020 statement. J. Clin. Epidemiol..

[14] Hořejší B., Kantorová L., Kantor J., Svobodová Z., Mareckova J. Adaptation of Music Therapists´ Practice to COVID-19 Pandemic: Going Virtual: A Scoping Review Protocol. https://osf.io/y948u/.

[15] Doraiswamy S., Abraham A., Mamtani R., Cheema S. (2020). Use of Telehealth During the COVID-19 Pandemic: Scoping Review. J. Med. Int. Res..

[16] Sasangohar F., Bradshaw M.R., Carlson M.M., Flack J.N., Fowler J.C., Freeland D., Head J., Marder K., Orme W., Weinstein B. (2020). Adapting an Outpatient Psychiatric Clinic to Telehealth During the COVID-19 Pandemic: A Practice Perspective. J. Med. Int. Res..

[17] Knott D., Block S. (2020). Virtual Music Therapy: Developing New Approaches to Service Delivery. Music Ther. Perspect..

[18] (2020). The Enclave at Round Rock Senior Living Hosts a Patio Music Party with the Help of North Austin Music Therapy. PR Newswire.

[19] (2020). College of Music to Continue Successful Virtual Tele-Therapy Services. US Fed News Service Including US State News.

[20] Negrete B. (2020). Family Matters. Meeting the Challenges of the COVID-19 Pandemic: Virtual Developmental Music Therapy Class for Infants in the Neonatal Intensive Care Unit. Pediatric Nurs..

[21] Little R. (2020). Music School’s Virtual Approach Proves a Hit. Aberdeen Evening Express.

[22] Gupta N. (2020). Singing Away the Social Distancing Blues: Art Therapy in a Time of Coronavirus. J. Humanist. Psychol..

[23] Berman A. Music Connects Us. 2020. Music Connects Us: The Use of Music and the Role of Music Therapy during a Pandemic Crisis Situation EMTC. Emtc-eu.com.

[24] Therapy N.L.M. Music Therapy in the Time of COVID—How NLMT’s Remote Sessions Actually Work. https://www.northlondonmusictherapy.com/post/music-therapy-in-the-time-of-covid-how-nlmt-s-remote-sessions-actually-work.

[25] Baker F., Krout R. (2009). Songwriting via Skype: An Online Music Therapy Intervention to Enhance Social Skills in an Adolescent Diagnosed with Asperger’s Syndrome. Br. J. Music Ther..

[26] Fuller A.M., McLeod R.G. (2019). The Connected Music Therapy Teleintervention Approach (CoMTTA) and its application to family-centred programs for young children with hearing loss. Aust. J. Music Ther..

[27] Bronson H., Vaudreuil R., Bradt J. (2018). Music Therapy Treatment of Active Duty Military: An Overview of Intensive Outpatient and Longitudinal Care Programs. Music Ther. Perspect..

[28] Levy C.E., Spooner H., Lee J.B., Sonke J., Myers K., Snow E. (2018). Telehealth-based creative arts therapy: Transforming mental health and rehabilitation care for rural veterans. Arts Psychother..

[29] Lightstone A.J., Bailey S.K., Voros P. (2015). Collaborative music therapy via remote video technology to reduce a veteran’s symptoms of severe, chronic PTSD. Arts Health.

[30] Gúth A.J., Gúth A.S. (2020). Music therapy in rehabilitation. Rehabilitácia.

[31] Schünemann H.J., Zhang Y., Oxman A.D. (2019). Distinguishing opinion from evidence in guidelines. BMJ.

[32] Shore J.H., Yellowlees P., Caudill R., Johnston B., Turvey C., Mishkind M., Krupinski E., Myers K., Shore P., Kaftarian E. (2018). Best practices in videoconferencing-based telemental health April 2018. Telemed. E-Health.

[33] Smith K., Ostinelli E., Macdonald O., Cipriani A. (2020). Covid-19 and telepsychiatry: Development of evidence-based guidance for clinicians. Jmir Ment. Health.

[34] Schünemann H.J. (2013). GRADE Handbook. http://gdt.guidelinedevelopment.org/app/handbook/handbook.html.

