# Supplementary material for: Adaptation of Music Therapists’ Practice to the Outset of the COVID-19 Pandemic—Going Virtual: A Scoping Review
Source: Int J Environ Res Public Health. 2021 May 12;18(10):5138. doi: 10.3390/ijerph18105138 (PMC8151825; doi:10.3390/ijerph18105138)
Supplement: Supplementary file 1 [file ijerph-18-05138-s001.zip › Supplementary S2 Search strategies, sources and results.pdf]

Databases		Number of duplicates	Fulltexscreening	Included in scoping review
**Scopus**	**8**			
**Web of Science**	**5**			
**CINAHL**	**5**			
**Medline**	**2**			
**ProQuest**	**93**			
**PubMed**	**4**			
**EMBASE**	**4**			
**PsycINFO**	**0**	**14**	**15**	**8**
**Cochrane**	**3**			
**DART**	**0**			
**Open Access**	**16**			
**Open Gr**	**0**			
**Electronic Theses Online Service**	**0**			
**Clinical trials & Current Controlled trials**	**0**			
**Google Scholar**	**58**			
	**198**	**14**	**15**	**8**
**Manual search**				
**Journals**	**11**	**4**	**7**	**0**
**Websites (adaptation articles)**	**-**	**-**	**3**	**2**
**Reference lists of included texts and 1 scoping review reference list**	**-**	**-**	**-**	**0**
				**2**
**Total**				**10**

**Journals searched manually, November 2020<br>Results: 11 (after screening title/abstract)<br>All results were duplicates or irrelevant**
Journal of music therapy
Nordic journal of music therapy
Music therapy perspectives
Voices
Approaches: An Interdisciplinary Journal of Music Therapy
Canadian music therapy journal
The New Zeland Journal of Music Therapy
Australian Journal of Music Therapy
Arts in Psychotherapy
Oxford University Press
La Revue française de Musicothérapie
MUSIQUE – THÉRAPIE – COMMUNICATION
REVUE DE MUSICOTHÉRAPIE
Musiktherapeutische Umschau (MU)
Musik und Gesundsein
Journal of Arts Therapies (JAT)
Quaderni italiani di Musicoterapia
https://mmd.iammonline.com/index.php/musmed/article/view/795
Japanese Journal of Music Therapy

**Websites of MT associations searched, November 2020**	
**USA**	musictherapy.org
UK	bamt.org

Europe	emtc-eu.com
Switzerland	Musictherapy.ch
Germany	Musiktherapie.de
New Zealand	Musictherapy.org.nz
World	WFMT
International	the International Association for Music & Medicine
Italy	https://musicaterapia.it/
Australia	Australian Musíc Therapy Association
Israel	YAHAT
Japan	Japanese

Scopus

Date of search 25.10.2020, 7:42-8:13

#	search	results
1	music therap* Ti/Ab/Key	654
2	"severe acute respiratory syndrome*" Ti/Ab/Key	17802
3	(outbreak* OR wildlife* OR pandemic* OR epidemic*) W/1 (China* OR Chinese* OR Huanan*)	533
4	(respiratory* W/2 (symptom* OR disease* OR illness* OR condition*) OR "seafood market*"	
	OR "food market*") W/10 (Wuhan* OR Hubei* OR China* OR Chinese* OR Huanan*) Ti/Ab/Key	248
5	(corona* OR corono*) W/1 (virus* OR viral* OR virinae*) Ti/Ab/Key	1547
6	(coronavirus* OR coronovirus* OR coronavirinae* OR Coronavirus* OR Coronovirus* OR	
	Wuhan* OR Hubei* OR Huanan OR "2019-nCoV" OR 2019nCoV OR nCoV2019 OR "nCoV-2019"	
	OR "COVID-19" OR COVID19 OR CORVID-19 OR CORVID19 OR "WN-CoV" OR WNCoV OR HCoV-19	
	OR HCoV19 OR CoV OR "2019 novel*" OR Ncov OR "n-cov" OR SARS-CoV-2 OR "SARSCoV-2" OR	
	"SARSCoV2" OR "SARS-CoV2" OR SARSCov19 OR SARS-Cov19 OR "SARSCov-19" OR "SARS-Cov-19"	
	OR Ncovor OR Ncorona* OR Ncorono* OR NcovWuhan* OR NcovHubei* OR NcovChina* OR	
	NcovChinese*) Ti/Ab/Key	72976
7	2-6 OR	73240
8	telemedicine Ti/Ab/Key	5002
9	telehealth Ti/Ab/Key	2438
10	internet Ti/Ab/Key	35631
11	online Ti/Ab/Key	56039
12	virtual Ti/Ab/Key	25814
13	distanc* Ti/Ab/Key	66578
14	internet-based (internet based) Ti/Ab/Key	1069
15	home-based (home based) Ti/Ab/Key	1423
16	audio-visual (audio visual) Ti/Ab/Key	634
17	web-based (web based) Ti/Ab/Key	5109
18	remote Ti/Ab/Key	27071
19	8-18 OR	202553
20	1 AND 7 AND 19	**8**

Web of Science Core Collection
Date of search 25.10.2020, 17:33-17:56

#	search	results
1	music therap* Ti/Topic	412
2	severe acute respiratory syndrome* Ti/Topic	5180
3	(outbreak* OR wildlife* OR pandemic* OR epidemic*) NEAR/1 (China* OR Chinese* OR Huanan*) Ti/Topic	370
4	(respiratory* NEAR/2 (symptom* OR disease* OR illness* OR condition*) OR "seafood market*"	
	OR "food market*") NEAR/10 (Wuhan* OR Hubei* OR China* OR Chinese* OR Huanan*) Ti/Topic	180
5	(corona* OR corono*) NEAR/1 (virus* OR viral* OR virinae*) Ti/Topic	699
6	(coronavirus* OR coronovirus* OR coronavirinae* OR Coronavirus* OR Coronovirus* OR Wuhan* OR	
	Hubei* OR Huanan OR "2019-nCoV" OR 2019nCoV OR nCoV2019 OR "nCoV-2019" OR "COVID-19"	
	OR COVID19 OR CORVID-19 OR CORVID19 OR "WN-CoV" OR WNCoV OR HCoV-19 OR HCoV19 OR CoV	
	OR "2019 novel*" OR Ncov OR "n-cov" OR SARS-CoV-2 OR "SARSCoV-2" OR "SARSCoV2" OR "SARS-CoV2"	
	OR SARSCov19 OR SARS-Cov19 OR "SARSCov-19" OR "SARS-Cov-19" OR Ncovor OR Ncorona* ORNcorono*	
	OR NcovWuhan* OR NcovHubei* OR NcovChina* OR NcovChinese*) Ti/Topic	43160
7	2-6 OR	43620
8	telemedicine Ti/Topic	2098
9	telehealth Ti/Topic	1211
10	internet Ti/Topic	15632
11	online Ti/Topic	28215
12	virtual Ti/Topic	13306
13	distanc* Ti/Topic	42078
14	internet-based Ti/Topic	671
15	internet based Ti/Topic	8552
16	home-based Ti/Topic	1063
17	home based Ti/Topic	6785
18	audio-visual Ti/Topic	274
19	audio visual Ti/Topic	505
20	web-based Ti/Topic	2956
21	web based Ti/Topic	8540
22	remote Ti/Topic	14747
23	8-22 OR	119643
24	1 AND 7 AND 23	**5**

ProQuest Central
Date of search 25.10.2020, 18:05-18:32

#	search	results
1	music therap* Ti/Ab/Main Subject	1254
2	severe acute respiratory syndrome* Ti/Topic	136464
3	(outbreak* OR wildlife* OR pandemic* OR epidemic*) N/1 (China* OR Chinese* OR Huanan*) Ti/Ab/Main Subject	2676
4	(respiratory* N/2 (symptom* OR disease* OR illness* OR condition*) OR "seafood market*"	
	OR "food market*") N/10 (Wuhan* OR Hubei* OR China* OR Chinese* OR Huanan*) Ti/Ab/Main Subject	443
5	(corona* OR corono*) N/1 (virus* OR viral* OR virinae*) Ti/Ab/Main Subject	6848
6	(coronavirus* OR coronovirus* OR coronavirinae* OR Coronavirus* OR Coronovirus* OR Wuhan* OR	
	Hubei* OR Huanan OR "2019-nCoV" OR 2019nCoV OR nCoV2019 OR "nCoV-2019" OR "COVID-19"	
	OR COVID19 OR CORVID-19 OR CORVID19 OR "WN-CoV" OR WNCoV OR HCoV-19 OR HCoV19 OR CoV	
	OR "2019 novel*" OR Ncov OR "n-cov" OR SARS-CoV-2 OR "SARSCoV-2" OR "SARSCoV2" OR "SARS-CoV2"	
	OR SARSCov19 OR SARS-Cov19 OR "SARSCov-19" OR "SARS-Cov-19" OR Ncovor OR Ncorona* ORNcorono*	
	OR NcovWuhan* OR NcovHubei* OR NcovChina* OR NcovChinese*) Ti/Ab/Main Subject	5353355
7	2-6 OR	5354233
8	telemedicine Ti/Ab/Main Subject	32785
9	telehealth Ti/Ab/Main Subject	6770
10	internet Ti/Ab/Main Subject	257767
11	online Ti/Ab/Main Subject	315603
12	virtual Ti/Ab/Main Subject	158683
13	distanc* Ti/Ab/Main Subject	343857
14	internet-based Ti/Ab/Main Subject	981
15	internet based Ti/Ab/Main Subject	10984
16	home-based Ti/Ab/Main Subject	2543
17	home based Ti/Ab/Main Subject	12198
18	audio-visual Ti/Ab/Main Subject	1622
19	audio visual Ti/Ab/Main Subject	2260
20	web-based Ti/Ab/Main Subject	4478
21	web based Ti/Ab/Main Subject	10313
22	remote Ti/Ab/Main Subject	69689
23	8-22 OR	1117082
24	1 AND 7 AND 23	**93**

PsycINFO/PsycARTICLES
Date of search 25.10.2020, 19:21-19:40

#	search	results
1	music therap* Ti/Ab/Subject term	101
2	severe acute respiratory syndrome* Ti/Subject term	89
3	(outbreak* OR wildlife* OR pandemic* OR epidemic*) N1 (China* OR Chinese* OR Huanan*) Ti/Ab/Subject tern	43
4	respiratory* N2 (symptom* OR disease* OR illness* OR condition*) OR "seafood market*"	
	OR "food market*") N10 (Wuhan* OR Hubei* OR China* OR Chinese* OR Huanan*) Ti/Ab/Main Subject	4
5	(corona* OR corono*) N/1 (virus* OR viral* OR virinae*) Ti/Ab/Subject term	30
6	(coronavirus* OR coronovirus* OR coronavirinae* OR Coronavirus* OR Coronovirus* OR Wuhan* OR	
	Hubei* OR Huanan OR "2019-nCoV" OR 2019nCoV OR nCoV2019 OR "nCoV-2019" OR "COVID-19"	
	OR COVID19 OR CORVID-19 OR CORVID19 OR "WN-CoV" OR WNCoV OR HCoV-19 OR HCoV19 OR CoV	
	OR "2019 novel*" OR Ncov OR "n-cov" OR SARS-CoV-2 OR "SARSCoV-2" OR "SARSCoV2" OR "SARS-CoV2"	
	OR SARSCov19 OR SARS-Cov19 OR "SARSCov-19" OR "SARS-Cov-19" OR Ncovor OR Ncorona* ORNcorono*	
	OR NcovWuhan* OR NcovHubei* OR NcovChina* OR NcovChinese*) Ti/Ab/Subject term	2419
7	2-6 OR	2503
8	telemedicine Ti/Ab/Main Subject	336
9	telehealth Ti/Ab/Main Subject	260
10	internet Ti/Ab/Main Subject	1465
11	online Ti/Ab/Main Subject	6900
12	virtual Ti/Ab/Main Subject	1094
13	distanc* Ti/Ab/Main Subject	2102
14	internet-based Ti/Ab/Main Subject	188
15	internet based Ti/Ab/Main Subject	236
16	home-based Ti/Ab/Main Subject	175
17	home based Ti/Ab/Main Subject	290
18	audio-visual Ti/Ab/Main Subject	67
19	audio visual Ti/Ab/Main Subject	85
20	web-based Ti/Ab/Main Subject	503
21	web based Ti/Ab/Main Subject	481
22	remote Ti/Ab/Main Subject	431
23	8-22 OR	11375
24	1 AND 7 AND 23	**0**

Ovid MEDLINE(R) 1946 to October 23, 2020

Date of search 25.10.2020, 19:41-19:55

#	search	results
1	Coronavirus/	3892
2	"severe acute respiratory syndrome*" Ti/Subject term	8036
3	((outbreak* or wildlife* or pandemic* or epidemic*) adj1 (China* or Chinese* or Huanan*)) Ti/Ab/Keyword Heading	196
4	respiratory* N2 (symptom* OR disease* OR illness* OR condition*) OR "seafood market*"	
	OR "food market*") N10 (Wuhan* OR Hubei* OR China* OR Chinese* OR Huanan*) Ti/Ab/Keyword Heading	478
5	((corona* or corono*) adj1 (virus* or viral* or virinae*)Ti/Ab/Keyword heading	1124
6	(coronavirus* OR coronovirus* OR coronavirinae* OR Coronavirus* OR Coronovirus* OR Wuhan* OR	
	Hubei* OR Huanan OR "2019-nCoV" OR 2019nCoV OR nCoV2019 OR "nCoV-2019" OR "COVID-19"	
	OR COVID19 OR CORVID-19 OR CORVID19 OR "WN-CoV" OR WNCoV OR HCoV-19 OR HCoV19 OR CoV	
	OR "2019 novel*" OR Ncov OR "n-cov" OR SARS-CoV-2 OR "SARSCoV-2" OR "SARSCoV2" OR "SARS-CoV2"	
	OR SARSCov19 OR SARS-Cov19 OR "SARSCov-19" OR "SARS-Cov-19" OR Ncovor OR Ncorona* ORNcorono*	
	OR NcovWuhan* OR NcovHubei* OR NcovChina* OR NcovChinese*) Ti/Ab/Keyword Heading	46141
7	2-6 OR	48845
8	telemedicine Ti/Ab/Keyword heading	10688
9	telehealth Ti/Ab/Keyword heading	4036
10	internet Ti/Ab/Keyword heading	42432
11	online Ti/Ab/Keyword heading	87667
12	virtual Ti/Ab/Keyword heading	46339
13	distanc* Ti/Ab/Keyword heading	213615
14	internet-based Ti/Ab/Keyword heading	7077
15	home-based Ti/Ab/Keyword heading	8677
16	audio-visual Ti/Ab/Keyword heading	1579
17	web-based Ti/Ab/Keyword heading	24872
18	remote Ti/Ab/Keyword heading	55333
19	8-18 OR	462006
20	music therap* Ti/Ab/Keyword Heading	2062
21	7 AND 19 AND 20	**2**

CINAHL Plus with Full Text
Date of search 25.10.2020, 19:57-20:18

#	search	results
1	(MH "Coronavirus") Major Subject Heading	859
2	"severe acute respiratory syndrome*" Ti/Ab/MH	3466
3	((outbreak* or wildlife* or pandemic* or epidemic*) N1 (China* or Chinese* or Huanan*)) Ti/Ab/MH	3
4	((respiratory* N2 (symptom* OR disease* OR illness* OR condition*) OR "seafood market*"	
	OR "food market*") N10 (Wuhan* OR Hubei* OR China* OR Chinese* OR Huanan*)) Ti/Ab/MH	157
5	((corona* or corono*) adj1 (virus* or viral* or virinae*)) Ti/Ab/MH	291
6	(coronavirus* OR coronovirus* OR coronavirinae* OR Coronavirus* OR Coronovirus* OR Wuhan* OR	
	Hubei* OR Huanan OR "2019-nCoV" OR 2019nCoV OR nCoV2019 OR "nCoV-2019" OR "COVID-19"	
	OR COVID19 OR CORVID-19 OR CORVID19 OR "WN-CoV" OR WNCoV OR HCoV-19 OR HCoV19 OR CoV	
	OR "2019 novel*" OR Ncov OR "n-cov" OR SARS-CoV-2 OR "SARSCoV-2" OR "SARSCoV2" OR "SARS-CoV2"	
	OR SARSCov19 OR SARS-Cov19 OR "SARSCov-19" OR "SARS-Cov-19" OR Ncovor OR Ncorona* ORNcorono*	
	OR NcovWuhan* OR NcovHubei* OR NcovChina* OR NcovChinese*) Ti/Ab/MH	24648
7	(MH "COVID-19") Major Subject Heading	9533
8	2-7 OR	27483
9	telemedicine Ti/Ab/MH	11290
10	telehealth Ti/Ab/MH	8265
11	internet Ti/Ab/MH	47973
12	online Ti/Ab/MH	71712
13	virtual Ti/Ab/MH	18541
14	distanc* Ti/Ab/MH	41315
15	internet-based Ti/Ab/MH	4514
16	home-based Ti/Ab/MH	6990
17	audio-visual Ti/Ab/MH	547
18	web-based Ti/Ab/MH	15218
19	remote Ti/Ab/MH	16435
20	9-19 OR	207904
21	music therap* Ti/Ab/MH	5197
22	7 AND 19 AND 20	**5**

EMBASE

Date of search 25.10.2020, 20:19-20:36

#	search	results
1	coronavirinae'/mj (Major Subject Heading)	5062
2	"severe acute respiratory syndrome*" Ti/Ab/Key	12258
3	((outbreak* or wildlife* or pandemic* or epidemic*) NEAR/1 (China* or Chinese* or Huanan*)) Ti/Ab/Key	108
4	((respiratory* NEAR/2 (symptom* OR disease* OR illness* OR condition*) OR "seafood market*"	
	OR "food market*") NEAR/10 (Wuhan* OR Hubei* OR China* OR Chinese* OR Huanan*)) Ti/Ab/Key	0
5	((corona* or corono*) NEAR/1 (virus* or viral* or virinae*)) Ti/Ab/Key	283
6	(coronavirus* OR coronovirus* OR coronavirinae* OR Coronavirus* OR Coronovirus* OR Wuhan* OR	
	Hubei* OR Huanan OR "2019-nCoV" OR 2019nCoV OR nCoV2019 OR "nCoV-2019" OR "COVID-19"	
	OR COVID19 OR CORVID-19 OR CORVID19 OR "WN-CoV" OR WNCoV OR HCoV-19 OR HCoV19 OR CoV	
	OR "2019 novel*" OR Ncov OR "n-cov" OR SARS-CoV-2 OR "SARSCoV-2" OR "SARSCoV2" OR "SARS-CoV2"	
	OR SARSCov19 OR SARS-Cov19 OR "SARSCov-19" OR "SARS-Cov-19" OR Ncovor OR Ncorona* ORNcorono*	
	OR NcovWuhan* OR NcovHubei* OR NcovChina* OR NcovChinese*) Ti/Ab/Key	64701
7	coronavirus disease 2019'/mj (Major Subject Heading)	48693
8	2-7 OR	76800
9	telemedicine Ti/Ab/Key	19079
10	telehealth Ti/Ab/Key	7320
11	internet Ti/Ab/Key	74231
12	online Ti/Ab/Key	207029
13	virtual Ti/Ab/Key	81263
14	distanc* Ti/Ab/Key	327955
15	internet-based Ti/Ab/Key	11794
16	home-based Ti/Ab/Key	14962
17	audio-visual Ti/Ab/Key	2705
18	web-based Ti/Ab/Key	44313
19	remote Ti/Ab/Key	91896
20	9-19 OR	806116
21	music therap* Ti/Ab/MH	3965
22	7 AND 19 AND 20	**4**

PubMed

Date of search 25.10.2020, 20:37-20:53

#	search	results
1	"Coronavirus"[Mesh] (Major Subject Heading)	38610
2	"severe acute respiratory syndrome*" Ti/Ab	12695
3	((outbreak* or wildlife* or pandemic* or epidemic*) N/1 (China* or Chinese* or Huanan*)) Ti/Ab	55
4	((respiratory* NEAR/2 (symptom* OR disease* OR illness* OR condition*) OR "seafood market*"	
	OR "food market*") NEAR/10 (Wuhan* OR Hubei* OR China* OR Chinese* OR Huanan*)) Ti/Ab	3
5	((corona* or corono*) N/1 (virus* or viral* or virinae*)) Ti/Ab	69
6	(coronavirus* OR coronovirus* OR coronavirinae* OR Coronavirus* OR Coronovirus* OR Wuhan* OR	
	Hubei* OR Huanan OR "2019-nCoV" OR 2019nCoV OR nCoV2019 OR "nCoV-2019" OR "COVID-19"	
	OR COVID19 OR CORVID-19 OR CORVID19 OR "WN-CoV" OR WNCoV OR HCoV-19 OR HCoV19 OR CoV	
	OR "2019 novel*" OR Ncov OR "n-cov" OR SARS-CoV-2 OR "SARSCoV-2" OR "SARSCoV2" OR "SARS-CoV2"	
	OR SARSCov19 OR SARS-Cov19 OR "SARSCov-19" OR "SARS-Cov-19" OR Ncovor OR Ncorona* ORNcorono*	
	OR NcovWuhan* OR NcovHubei* OR NcovChina* OR NcovChinese*) Ti/Ab	82283
7	"COVID-19" [Supplementary Concept] (Major Subject Heading)	31531
8	2-7 OR	89266
9	telemedicine Ti/Ab	14316
10	telehealth Ti/Ab	5998
11	internet Ti/Ab	54823
12	online Ti/Ab	146638
13	virtual Ti/Ab	62494
14	distanc* Ti/Ab	286099
15	internet-based Ti/Ab	8728
16	home-based Ti/Ab	10835
17	audio-visual Ti/Ab	2182
18	web-based Ti/Ab	31543
19	remote Ti/Ab	72739
20	9-19 OR	639923
21	music therap* Ti/Ab	2553
22	7 AND 19 AND 20	**4**

Cochrane Library

(covid OR coronavirus) AND (music therap*) AND (online OR virtual OR home-based OR remote OR telemedicine OR telehealth)

Results: 3

https://clinicaltrials.gov/ct2/show/NCT04537858 (dostupný z Clinical trial) == irrelevant

https://clinicaltrials.gov/ct2/show/NCT04329533 (dostupný z Clinical trial) == irrelevant

https://pubmed.ncbi.nlm.nih.gov/32665041/ (dostupný z PubMed) == duplicate

Open Access Theses and Dissertations

(covid OR coronavirus) AND (music therap*) AND (online OR virtual OR home-based OR remote OR telemedicine OR distanc* OR telehealth)

Results: 16

Google Scholar

(covid OR coronavirus) AND (music therap*) AND (online OR virtual OR home-based OR remote OR telemedicine OR distanc* OR telehealth)

Results: 58
