# Supplementary material for: Adaptation of Music Therapists’ Practice to the Outset of the COVID-19 Pandemic—Going Virtual: A Scoping Review
Source: Int J Environ Res Public Health. 2021 May 12;18(10):5138. doi: 10.3390/ijerph18105138 (PMC8151825; doi:10.3390/ijerph18105138)
Supplement: Supplementary file 1 [file ijerph-18-05138-s001.zip › Supplementary S3 Data charting tables for individual papers and a summary of data table.pdf]

Table S15. Data charting tables for individual papers and a summary of data table.

No.	Author, date, country, design, type of text	Setting and client group	Adaptations	Examples of music therapy activities	Benefits/challenges
1	The Enclave at Round Rock Senior Living Hosts a Patio Music Party with the Help of North Austin Music Therapy<br><br>March 2020<br>USA<br><br>Newspaper article	Retirement home<br><br>Seniors, aging adults, ASD, developmental challenges and neurological diseases and injuries	Adaptation of the form and goals: Therapeutic goals were focused not only on residents, but also on team members and community needs. Therapists wanted residents and team members to focus on what they can do and not what we they can't do, on feelings of happiness, social, emotional and physical connection through music.<br>Adaptation concerning the space – sessions took place on balcony.<br>Adaptation of musical instruments: Using ordinary things for music creation.	Residents were provided with a plastic Easter egg and tasked to filling it with things from their apartment to create a noisemaker. The concert then took place on a patio 2 times for one hour to rotate the sides of the buildings. Residents were in their balconies. Senior community singing and dancing, all while maintaining appropriate social distancing.	Practice music therapy even if it means maintaining social distancing or staying outdoors.
2	Music School's Virtual Approach Proves a Hit<br><br>June 2020<br><br>Newspaper article	Educational setting<br><br>Regular students and students who need additional support	Adaptation of musical instruments: Using ordinary things for music creation.<br>Adaptation of the form of therapy: Making weekly 10 minute videos for children and posting them on Youtube. Incorporating MAKATON into musical videos.<br>Adaptation of goals: What we hope to achieve by producing these episodes is accessibility for everyone at home to make music, have fun and still be creative. We want to promote that anyone can make music and out of anything and that it can be done in this fun and creative way.	Household items to create unique melodies and invites kids to get involved throughout. Things we can find around the household - sweetie tins, plastic bottles, pots and pans, spoons and more are used throughout the sessions.	
3	College of Music to Continue Successful Virtual Tele-therapy Services	Educational setting	Adaptation in the training programs of music therapists: Switching to teleservices also	Active music making, movement to music, song-writing and song discussion.	This new type of working with clients offers opportunities and challenges for student training

	August 2020<br>USA<br>Case report<br>Newspaper article	Students and young adults with disabilities	provided the opportunity for us to rethink current practice and expand how we prepare students for practice moving forward.		and teaches them how to be flexible music therapists.<br>I feel like we gained a lot from doing this online, rather than losing anything<br>It is an incredible alternative for in-person clinical hours and really works, both for us as students and for the clients. Using tele-services is can be beneficial to some clients due to the easy access.
4	**. North London music therapy**<br><br>Music Therapy in the Time of COVID - How NLMT's Remote Sessions Actually Work<br><br>Website article	Music therapy session within North London music therapy organisation<br><br>Music therapists and their clients in online therapy	Adaptation of technology:<br>You [client] have to rely on a good internet connection; sometimes there are breaks in the line, or weird sounds; sometimes you can't quite hear what your therapist said or played; sometimes your therapist can't quite hear you; sometimes the call gets disconnected. Think about your sound set-up. Laptop is preferable to phone. Using microphone or even an audio interface is preferable.<br>Adaptation related to space:<br>You [client] need to consider the room you're in quite carefully - quiet, so not much background noise, and that's unlikely to be disturbed for the duration of your session. Check whether you're happy that what you have in the background can be seen by your therapist.	We don't need instruments necessarily for music therapy; suggestions: can the client get some instruments, make some, could something be used as makeshift percussion, drumming surfaces in the room, singing.<br>We've found so far that the best method of music making online is by using free improvisation.	It feels weird [for the client] at first.<br>One of the most difficult things about remote music making is that it can feel very isolating [for the client].<br>Our patients are pleased to be able to have the option to carry therapy on in some form.
5	Berman<br>EMTC<br><br>Music Connects Us<br><br>September 2020	Music therapy association<br><br>Music therapists and	Music therapists can offer remote sessions over the phone or online, using secure software programs. There must be a clear privacy policy	Song writing and composing music:<br>• Adding lyrics to instrumental music or loops.<br>• Receptive music therapy, including sharing	Music therapists offer this service to ensure continued support, and it is not intended as a permanent alternative to 'live' sessions. In online sessions, it may not be possible to offer

	Flyer[1]	music therapy clients	regarding the choice of program and the way it is used.	playlists or watching videos together.<br>• Turn taking – making musical dialogues.<br>• Singing and accompanying vocals while one microphone is turned off.<br>• Reflecting verbally on the music made and feelings that arise during the crisis.	music therapy exactly as usual. Making music online is often difficult because of the delay and poor sound quality, but there are possibilities that can work well.
6	Gupta<br><br>Singing Away the Social Distancing Blues: Art Therapy in a Time of Coronavirus<br><br>September 2020<br><br>Case report<br>Essay by a psychology professor	Educational setting<br><br>University (undergraduate art therapy program)	Adaptation of instruments: using of ordinary things to make a sound and any instruments found in home environment<br>Adaptations of goals: To show how music relieves stress and brings joy and harmony during the pandemic, how it reminds people they are not alone.	Improvisational virtual jam session using Zoom. Each student was invited to bring an object that can serve as an instrument, be it a guitar or harmonica or spoons or their voice.	"We sang together, clapped together, strummed together, and laughed together, accessing a primal mode of communal being-together through collective sound making across our individual screens."<br>The rhythm was slightly delayed because of the Internet's lag-time.<br>Harmony could not find perfect synchronization through our laptops and phones.<br>It brought us enough joy and love to overpower the terror of COVID-19 for just a little while<br>There is a profound desire to protect each other, sacrifice for each other, and be together-in-solidarity as an interconnected, global community when faced with this grave threat to the human species. Our lives depend quite literally on our ability to love each other—across borders, balconies, and computer screens.
7	Sasangohar	An outpatient psychiatric clinic	Adaptations regarding technology: Essential to leverage multiple platforms and modalities	"One client became anxious and frustrated by initial difficulties downloading Webex; therefore, we pivoted to using	Both verbal and non-verbal communication is disrupted when wearing a mask and keeping distance of 2m. Masks can

[1] Available at: https://www.emtc-eu.com/news/2020/9/2/music-connects-us-the-use-of-music-and-the-role-of-music-therapy-during-a-pandemic-crisis-situation

Adapting an Outpatient Psychiatric Clinic to Telehealth During the COVID-19 Pandemic: A Practice Perspective<br>October 2020<br>Case study<br>Journal article	Patients with psychiatric disorders, interpersonal dysfunctions and problems, sleep disorders	to facilitate the initial transition<br>adapt a suitable system of work to different clients - choice of communication channel, hours, length, etc. Interruptions may disrupt session plans and require management. It is useful to wear headphones for each telepractice.<br>Make sure sessions are recorded in case the session is interrupted - especially in group therapies.<br>Logistical adaptations: necessary to incorporate additional consultation meetings between colleagues (therapists).<br>Maintain work-life balance by separation of space and time - with working from home environment come challenges of mixing work and personal life - try to find a working space at home, which is only used for work. It is essential to not call from a personal account.<br>Acknowledge that telepractice might be more demanding and time consuming due to adaptation to technical difficulties. Acknowledge that there is such a thing as zoom fatigue, therefore it is important to plan out the hours spent online wisely.<br>Adaptations of space: Setting the physical and visual backdrop - ensure the work setting looks tidy and professional.<br>Informing the client: Ensure they are comfortable and oriented in the technology.	FaceTime (Apple Inc), which did not require an additional tool and was familiar to the client." "Another client, who was feeling lonely and disconnected but struggled to find time for sessions because of childcare demands, was able to feel connected by occasionally texting her therapist."	also muffle voices and limit a therapist's assessment of patient affect.<br>Due to working from home setting, the relationship between patient and therapist is in risk of losing its boundaries. - Overall strengths of telepsychiatry include receptive and well-engaged responses from patients as well as the expansion of boundaries, which provides a directly contextualized view into patients' home lives.<br>Virtual Groups Have Been Well-Attended, and Engagement in Some Respects Has Increased<br>Patients are receptive and well-engaged - due to the isolation caused by pandemic, patients were enthusiastic about finding different channels of communication/provided therapy.<br>Telepsychiatry Expands the Boundaries of Psychological Intervention Into the Real World

			Adaptation of the form of therapy:<br>Adjust interviewing styles, such as avoiding talking over the patient - adjust to a bit of different style of therapy.		
8	Gaddy<br><br>COVID-19 and Music Therapists' Employment, Service Delivery, Perceived Stress, and Hope: A Descriptive Study<br><br>September 2020<br>USA<br><br>Cross sectional study (survey of music therapy professionals)<br><br>Journal article	N = 1,196 (music therapists)<br><br>Private Practice/Contractual: 37.38%; Schools: 24.80%; Hospice: 19.43%; Psychiatry: 19.00%<br><br>Autism Spectrum Disorder: 44.73%; Developmental disabilities: 44.02%; Older Adults: 35.85%; Alzheimer's: 34.35%	Virtual service delivery – the most frequent topic within open comments (25.49%).<br>MTs in private practice were impacted more than MTs in other settings<br>Respondents reporting changes in employment (n = 886) were most often providing remote clinical services from home (67.40%).	Among those who reported delivering alternative services compared to pre-pandemic: Telehealth services (54.81%), virtual music lessons (17.01%), prerecorded songs/playlists (16.98%), and prerecorded video sessions (16.00%).	Respondents discussing virtual services spoke more frequently about the benefits than the difficulties of such services, with many mentioning the lack of access to virtual service delivery options at the time of survey and some expressing mixed perspectives regarding this type of service delivery.<br>Regarding virtual services (the most frequent topic), some MTs expressed gratitude for the opportunity to continue earning money and providing services during this time of social distancing. Others expressed frustration that virtual service technology is difficult to learn, not appropriate for all clients, and cost prohibitive for many clients and clinicians. Additionally, MTs mentioned barriers and inequities, which kept clients from accessing these services<br>„Telehealth has, by necessity, become a way we can access people who are either in remote locations or who are among the very sick or immunocompromised."<br>"I've been impressed by the quick pivot to telehealth by the field. While this isn't ideal and doesn't replace face to face MT, it is providing service to the clients."

					“Standards have lowered even more in pretending that telehealth is effective.”<br>“MT doesn't translate well online so I feel very limited”<br>33.85% “I’m surprised to discover how much I can do via ZOOM. It’s forced me to be creative in ways I hadn’t been before.”<br>27 20.77% “The company I work for will not accept Telehealth.”<br>23 17.69% “Telehealth and learning how to handle technology has<br>been an uphill battle and constant struggle.”<br>12.31% “Telehealth is a great solution; however, not every client is appropriate for it.”
9	Knott<br><br>Virtual Music Therapy: Developing New<br>Approaches to Service Delivery<br><br>September 2020<br>USA<br><br>N/A<br><br>Journal article	Healthcare systems, educational setting and communities<br><br>Any client of music therapy	Authors created a THREE-TIERED SCAFFOLD MODEL for telepractice:<br>1.Curate Online Resources.<br>2.Create Original Content.<br>3.Implement Telehealth.<br><br>Adaptations related to technology: music therapists must consider which options best match the technical and physical capabilities of their clients and requirements of their payor. MT also must ensure that clients have suitable technology for telehealth (laptop or computer, video camera, microphone, additionally headphones, USB stick etc.)<br>Informing the client: help client to find a suitable space from which client will access the virtual music therapy	**Curate online resources**<br>Identifying preexisting content (audio, videos, and music-making instructions) readily available online that reinforce the therapeutic uses of music or social and emotional learning concepts.<br>Music therapists may create word documents or PDFs directed to patients and families, which outline online resource recommendations.<br>**Create original content and implement telehealth**<br>Finger play songs can be used to reinforce infants’ and toddlers’ motor and language development.<br>Preschool and early childhood music can be used to teach pre-academic and academic concepts.	

		(ideally from a quiet room, good light etc.)	Relaxation-oriented audio and video recordings may be created to support teens and adults in developing new coping skills. Example of a session: A hospitalized adolescent with COVID-19 on a behavioural health unit was engaged in a video teleconference session to reinforce coping skills using therapeutic music instruction. During the session, the patient complained of difficulty getting a deep breath (dyspnea). The music therapist engaged the patient in therapeutic singing, which resulted in the patient reporting improved ease of breathing at the end of the session. Child VMT for therapeutic play. A developmental music play session was conducted through video conferencing with a hospitalized toddler receiving cardiac care with the objective of increasing arm use, specifically reaching. The screen sharing function was utilized to facilitate the child reaching to touch the screen when prompted in the song. After the parent reported the child had touched the screen, the music therapist would continue the song, providing cause and effect reinforcement and sustaining engagement in the treatment. Adult VMT for anxiety. Utilizing an adult patient's bedside phone, a music-assisted relaxation intervention was facilitated in order to reduce	

				anxiety. The patient was led through a scripted relaxation while the therapist spoke and facilitated instrumental acoustic guitar music in the background. Throughout the session, the patient was introduced to guided imagery, breathing techniques, and mindfulness-based strategies. Additionally, the patient was provided resources for free online-guided imagery tracks along with education on regimen and usage with examples provided including preparing for anxiety-inducing events, such as medical procedures, discharge, or times of day when the patient desires deepened relaxation such as at bedtime.	
10	Negrete<br><br>Meeting the Challenges of the COVID-19 Pandemic: Virtual Developmental Music Therapy Class for Infants in the Neonatal Intensive Care Unit<br><br>July - August 2020<br>USA<br><br>Case report<br><br>Journal article	Hospital environment<br><br>Infants at Intensive Care Nursery and their parents/relatives	Adaptation of the helping staff roles: The online class also minimizes the amount of staff time required to help with the class because all the family or nurse needs to do is to log on. Identify at least one on-site staff member who can help families troubleshoot technical difficulties.	Music therapist facilitates the virtual session from home, Sessions is now held twice a week for 20 minutes over Zoom. Videoconference via Zoom, using a gallery view - for the infant, parents, other family members.<br>Techniques used have not changed:<br>Songs that introduced socialization.<br>Used singing and shakers.<br>Songs taught baby sign language.<br>Before each song, the music therapist would educate the parents on how to participate in the song with their child, providing hand-over-hand support, pointing to the different body parts the song was	Example of personal experience:<br>“Through Zoom, M. has been able to maintain one of his favourite therapies, which is music therapy class. He has found his loud voice with singing along with Brianna. He also recognizes the faces of other participants as well as Brianna. Outside of the class, we sing the songs from class, which he enjoys.”<br>“My son has truly found joy in music through our Zoom classes. He is moving his hands, smiling, paying attention to when the music stops or changes, and even shaking his shaker.”

		cuing, explaining how the song supported their infant's development, and demonstrating how the song could be used when not in a group setting.	They described many benefits enabling:<br>• for babies to see a face and mouth without a mask,<br>• more infants are able to attend at the same time,<br>• easier coordination of respiratory support and transportation, eg. for the infants with tracheostomies, or those on isolation<br>• decreased amount of staff time required to help with the sessions.<br>• Family members off site can attend.<br>• Interpreting services can connect easily.<br><br>Challenge:<br>Setting time limit for screen time. It should not exceed 25 minutes, and the screen should be turned off if the infant shows signs of over stimulation.

**Author, month+year, country**	**PR Newswire, March 2020, USA**
**Title**	**The Enclave at Round Rock Senior Living Hosts a Patio Music Party with the Help of North Austin Music Therapy**
Type of reference and language	Newspaper article, English
Setting and services	Retirement home for seniors
Target client group	Seniors, aging adults, autism, developmental challenges and neurological diseases or injuries.
Main objectives	To offer a social, emotional, and physical connection with one another through music
Any additional objectives	N/A
Methodology	Case report on a music therapy concert with adequate social distancing during COVID-19 pandemic
Participants/Population (definition, age, gender, profession, size and characteristics of the sample(s)	Seniors in a retirement home
Main outcomes	"It was an incredible experience to see everyone come together and contribute to the music. We want residents and team members to focus on what we can do and not what we can't do, so we utilized the nice weather and the apartment balconies to make this happen. The residents had a blast, and it was obvious that this is the kind of happiness the residents, our team members, and our communities need during this terrible crisis."
Additional outcomes	
Music therapeutic techniques	Live music therapy concert. Residents were provided with a plastic Easter egg and tasked to filling it with things from their apartment to create a noise maker. The concert then took place on a patio 2 times for one hour to rotate the sides of the buildings. Residents were in balconies.
Examples of work with concrete clients in the text	Senior community singing and dancing, all while maintaining appropriate social distancing.<br><br>Provided each resident with a plastic Easter egg and tasked them with filling it with things from their apartment to create a noise maker.
Any recommendations for music therapists´ practice in COVID-19 pandemic	
Any other recommendations	
Conclusions of the study	Social distancing should never mean social isolation.

Author, month+year, country	Little, June 2020, UK
**Title**	**Music School's Virtual Approach Proves a Hit**
Type of reference and language	Newspaper article, English
Setting and services	Educational setting. North east music school, providing interactive music classes, drumming and music tuition to students during the COVID 19 pandemic.
Target client group	Regular students and students who have additional support needs.
Main objectives	To describe the transfer of the "Trash Band" to a virtual setting.
Any additional objectives	
Methodology	Case report (NR)
Participants/Population (definition, age, gender, profession, size and characteristics of the sample(s)	N/A
Main outcomes	Every seven days, the MTs write a new script, create a new piece of music and film and edit a 10-minute video for their audience. It's all up on YouTube and the website. https://bit.ly/3eQZVK3
Additional outcomes	
Music therapeutic techniques	The Trash Band use a number of household items to create unique melodies and invites kids to get involved throughout. Things we can find around the household - sweetie tins, plastic bottles, pots and pans, spoons and more are used throughout the sessions.<br>Each clip features signing for children who have additional support needs.<br>Use of fun and creative music videos to get clients engaged.<br>Using MAKATON instead of British Sign Language for signing.
Examples of work with concrete clients in the text	
Any recommendations for music therapists´ practice in COVID-19 pandemic	
Any other recommendations	
Conclusions of the study	What we hope to achieve by producing these episodes is accessibility for everyone at home to make music, have fun and still be creative.<br>We want to promote that anyone can make music and out of anything and that it can be done in this fun and creative way taking the viewers on a Trash Band Adventure with us.

**Author, month+year, country**	**Florida State University, August 2020, USA**
**Title**	**College of Music to Continue Successful Virtual Tele-therapy Services**
Type of reference and language	News article, English
Setting and services	Florida State University College of Music, music therapy services provided by the faculty and its students
Target client group	Students and young adults with disabilities
Main objectives	Reporting on how music therapists (university students and faculty) provide telehealth due to the Covid 19 pandemic.
Any additional objectives	
Methodology	Case report on Florida State University College of Music's tele practice transition
Participants/Population (definition, age, gender, profession, size and characteristics of the sample(s)	N/A
Main outcomes	This new type of working with clients offers opportunities and challenges for student training and teaches them how to be flexible music therapists.<br>Switching to teleservices also provided the opportunity for us to rethink current practice and expand how we prepare students for practice moving forward.
Additional outcomes	It is an incredible alternative for in-person clinical hours and really works, both for us as students and for the clients. I feel like we gained a lot from doing this online, rather than losing anything.
Music therapeutic techniques	Virtual sessions still give participants the opportunity to engage in active music making, movement to music, songwriting and song discussion.
Examples of work with concrete clients in the text	MT with focus on client's life, social, independence and employability skills and include live music.
Any recommendations for music therapists´ practice in COVID-19 pandemic	
Any other recommendations	
Conclusions of the study	Using tele-services is an innovative form of offering therapy sessions, which can be beneficial to some clients due to the easy access. The Florida State University College of Music are rethinking the way MT was practiced until now

Author, month+year, country	North London music therapy, 2020, UK
Title	Music Therapy in the Time of COVID - How NLMT's Remote Sessions Actually Work
Type of reference and language	Website article, English
Setting and services	Music therapy organization
Target client group	N/A
Main objectives	To describe how MT services continue during the pandemic
Any additional objectives	
Methodology	N/A
Participants/Population (definition, age, gender, profession, size and characteristics of the sample(s)	NR
Main outcomes	It feels weird at first. This is to be expected. You're in a different room; so is your therapist; you don't feel the presence of someone else being there with you. You have to rely on a good internet connection; sometimes there are breaks in the line, or weird sounds; sometimes you can't quite hear what your therapist said or played; sometimes your therapist can't quite hear you; sometimes the call gets disconnected.<br>So much of music therapy is based on the connection between patient and therapist - and one of the most difficult things about remote music making is that it can feel very isolating. Sometimes when playing, it's possible to feel very aware of your own playing - a reminder that your therapist is not in the room with you. It's a new sensation, and might take a bit of getting used to.<br><br>You [client] need to consider the room you're in quite carefully. This would usually be up to your therapist to think about and organise for you. You need to make sure you're in a room that's quiet, so not much background noise, and that's unlikely to be disturbed for the duration of your session. Check whether you're happy that what you have in the background can be seen by your therapist.<br><br>Think about your sound set-up. When using a phone, especially on speaker phone, the sound your therapist makes will cut out when you're making sound. If you can, use a laptop instead of a phone, and use a pair of headphones to help you hear the sound. Even better, if you've got access to a microphone or even an audio interface, make sure they are compatible with your laptop and use them during your session for increased sound quality.
Additional outcomes	Our patients are pleased to be able to have the option to carry therapy on in some form
Music therapeutic techniques	Remote music making is a whole different ball game. Not every patient has instruments at home. That's ok, we don't need instruments necessarily for music therapy - but if you'd like some instruments, how can you get some, or make some? Is there something you could use as makeshift percussion? Would drumming surfaces in the room you're in feel satisfactory enough? Would you be happy to sing, even if you don't usually?<br>We've found so far that the best method of music making online is by using free improvisation.
Examples of work with concrete clients in the text	
Any recommendations for music therapists´ practice in COVID-19 pandemic	
Any other recommendations	
Conclusions of the study	

Author, month+year, country	Berman (EMTC), September 2020, Europe
**Title**	**Music Connects Us**
Type of reference and language	Flyer, English
Setting and services	N/A
Target client group	N/A
Main objectives	Introduce music therapy to potential clients during the pandemic
Any additional objectives	How to use music to promote well-being
Methodology	N/A
Participants/Population (definition, age, gender, profession, size and characteristics of the sample(s)	N/A
Main outcomes	Music therapists can offer remote sessions over the phone or online, using secure software programs. There must be a clear privacy policy regarding the choice of program and the way it is used. Music therapists offer this service to ensure continued support, and it is not intended as a permanent alternative to 'live' sessions. In online sessions, it may not be possible to offer music therapy exactly as usual. Making music online is often difficult because of the delay and poor sound quality, but there are possibilities that can work well.
Additional outcomes	
Music therapeutic techniques	Songwriting and composing music (a crisis can surprise us with creative impulses!)<br>• Adding lyrics to instrumental music or loops<br>• Receptive music therapy, including sharing playlists or watching videos together<br>• Turn-taking – making musical dialogues<br>• Singing and accompanying vocals while one microphone is turned off<br>• Reflecting verbally on the music made and feelings that arise during the crisis
Examples of work with concrete clients in the text	
Any recommendations for music therapists´ practice in COVID-19 pandemic	
Any other recommendations	
Conclusions of the study	

**Author, month+year, country**	**Gupta, September 2020, GA, USA**
**Title**	**Singing Away the Social Distancing Blues: Art Therapy in a Time of Coronavirus**
Source database, type of reference and language	Journal article, English
Setting and services	University (undergraduate art therapy program)
Target client group	N/A
Main objectives	To describe the adaptation of one music therapy undergraduate university class/lesson to a virtual setting (Zoom)
Any additional objectives	To show examples of how music relieves stress and brings joy and harmony during the pandemic, how it reminds people they are not alone.<br>Similar objectives for visual arts and cinema, not relevant for this paper.
Methodology	An essay by a psychology professor, case report
Participants/Population (definition, age, gender, profession, size and characteristics of the sample(s)	University students in an art therapy program (n=20)
Main outcomes/results	**social distancing did not impede our musical solidarity**<br>The rhythm was slightly delayed because of the Internet's lag-time.<br>Harmony could not find perfect synchronization through our laptops and phones.<br>We sang together, clapped together, strummed together, and laughed together, accessing a primal mode of communal being-together through collective sound making across our individual screens.<br>**brought us enough joy and love to overpower the terror of COVID-19 for just a little while**
Additional outcomes/results	
Music therapeutic techniques	Improvisational virtual jam session using Zoom. Each student was invited to bring an object that can serve as an instrument, be it a guitar or harmonica or spoons or their voice.
Examples of work with concrete clients in the text	none
Any recommendations for music therapists´ practice in COVID-19 pandemic	none
Any other recommendations	none
Conclusions of the study	There is a profound desire to protect each other, sacrifice for each other, and be together-in-solidarity as an interconnected, global community when faced with this grave threat to the human species. Our lives depend quite literally on our ability to love each other—across borders, balconies, and computer screens.

Author, month+year, country	Sasangohar et al, October 2020, USA
**Title**	**Adapting an Outpatient Psychiatric Clinic to Telehealth During the COVID-19 Pandemic: A Practice Perspective**
Type of reference and language	Journal article, English
Setting and services	An outpatient psychiatric clinic at Houston Methodist Hospital, established in January 2018<br>Functional Rehabilitation Program, intensive outpatient program, a modified functional rehabilitation program, individual psychotherapy, couples counseling, psychotropic medication management, and art and music therapies.
Target client group	Psychiatric patients (nonemergency) =outpatients<br>Depression, anxiety-related disorders,<br>trauma, psychosis, interpersonal dysfunction, chronic pain, sleep disorders, emotional regulation problems, suicidality.
Main objective of the paper	Summarize several important successful improvisations and challenges to support broader and more diverse adoption efforts in telepractice.<br>Explore the effects of new types of therapy on clients as well as the rapid adaptation to telemedicine by client and therapist.<br>Discuss the logistics around telepractice, learning to work with systems - which work best, which client prefers what, etc.
Any additional objectives	Art/music therapy group programming serves as an applied case study, demonstrating the value of a well-managed web-based program as well as the challenges.
Methodology	Case study - the clinic's experience when working with an art/music therapy group.
Participants/Population (definition, age, gender, profession, size and characteristics of the sample(s)	NR. Psychiatric clinical team and their clients
Main outcomes	Essential **to leverage multiple platforms and modalities** (eg, Cisco Webex, Microsoft Teams, email, telephone calls, the EHR, patient portal communications) **to facilitate the initial transition**.<br>**In the case of pandemic, telepractice is convenient since both verbal and non verbal communication is key in therapy, which is disrupted when wearing a mask and keeping distance of 2m. Masks can also muffle voices which limits the communication. Masks also limit a therapist´s assessment of patient affect.**<br>One size doesn't fit all in this case, therefore it is essential to **adapt a suitable system of work to different clients - choice of communication channel, hours, length, etc**.<br>Shelter-in-place restrictions challenge active interprofessional collaboration - **necessary to incorporate additional consultation meetings between colleagues (therapists**).<br>**Maintain work-life balance by separation of space and time - with working from home environment come challenges of mixing work and personal life - try to find a working space at home, which is only used for work**.<br>**Setting the physical and visual backdrop requires forethought and design - ensure the work setting - even if from home, looks tidy and professional**.<br>**Interruptions may disrupt session plans and will require management - sometimes interruptions are inevitable, especially in group therapies - ensure there is a video replay function.**<br>**Set the therapeutic frame with intentionality to manage risk - due to working from home setting**, the relationship between client and therapist is in risk of loosing its boundaries - ensure that this does not happen. **It is essential to not call from a personal account.**<br>Introduce the client to the virtual space and expect to take up initial session time - as this form of communication is novel to client, **ensure they are comfortable and oriented in the technology**. Therapists may be required to offer an initial 10-15 minutes introduction to technology for clients in order to feel confident and comfortable with the systems used.<br>**Virtual Groups Have Been Well-Attended, and**<br>**Engagement in Some Respects Has Increased**<br>**Patients are receptive and well-engaged - due to the isolation caused by pandemic, patients were enthusiastic about finding different channels of communication/provided therapy.**<br>**Telepsychiatry Expands the Boundaries of Psychological Intervention Into the Real Worl**d
Music therapeutic techniques	NONE
Examples of work with concrete clients in the text	One client became anxious and<br>frustrated by initial difficulties downloading Webex; therefore, we pivoted to using FaceTime (Apple Inc), which did not require<br>an additional tool and was familiar to the client.<br>Another client, who was feeling lonely and disconnected but struggled to find time for sessions because of childcare demands, was able to feel connected by occasionally texting her therapist.
Any recommendations for music therapists´ practice in COVID-19 pandemic	**Acknowledge that telepractice might be more demanding and time consuming due to adaptation to technical difficulties**.

	Be aware to not get too comfortable/personal/intimate due to home setting - maintain boundaries, **adjust their interviewing styles, such as avoiding talking over the patient - adjust to a bit of different style of therap**y.<br>**Acknowledge that there is such a thing as zoom fatigue, therefore it is important to plan out the hours spent online wisely**.<br>**Therapists should ensure to always call from the same environment with a professionally looking background to keep a professional relationship between them and their client**.
Any other recommendations	Be patient with adapting to this new system, understand that it is a process that gets better with practice and adaptation of both client and therapist.<br>**It is useful to wear headphones for each telepractice**, since they avoid possible distraction in the household.<br>**Make sure sessions are recorded in case the session is interrupted - especially in group therapies**.
Conclusions of the study	Closer observation of telepractice and sharing information = improvement in telepractice.<br>**The circumstances of the COVID-19 pandemic have created unprecedented challenges that are taxing the physical and mental health of the population; however, the pandemic has also created unprecedented opportunities to learn, teach, innovate, and evaluate telepsychiatry strategies as necessity spurs their adoption**.<br>Overall strengths of telepsychiatry include receptive and well-engaged responses from patients as well as the expansion of boundaries, which provides a directly contextualized view into patients' home lives.

**Author, month+year, country**	**Gaddy, September 2020, CO, USA**
**Title**	**COVID-19 and Music Therapists' Employment, Service Delivery, Perceived Stress, and Hope: A Descriptive Study**
Type of reference and language	Journal article, English
Setting and services	N/A (survey of music therapy professionals in USA)
Main objective of the paper	To determine the impact of the pandemic on the employment, service delivery, stress, and hope of music therapy professionals in the United States.
Any additional objectives	Additional open comments in the survey - allowing respondents to comment regarding their **experiences** during the pandemic
Methodology	A cross-sectional study. Mixed qualitative + quantitative. A survey consisted of 51 items within four sections: (1) Demographics, (2) Employment/Services Provided, (3) Levels of Hope, and (4) Levels of Stress. It was drafted by authors and validated externally. It was administered using Qualtrics beginning on April 7, 2020, and remained open for two weeks. It was sent via email from the Certification Board for Music Therapists (CBMT) roster, which included 8,080 MTs in the United States. The survey link was also posted on social media platforms such as Facebook and LinkedIn. The survey included two standard scales: The Adult Hope Scale and the Perceived Stress Scale-10 (PSS-10).
Participants/Population (definition, age, gender, profession, size and characteristics of the sample(s)	1,196 music therapists, strictly in USA<br>**Age**: 18–24: 10.45%; 25–34: 47.07%; 35–44: 18.81%; 45–54: 10.62%; 55–64: 8.95%; 65–74: 3.68%; 75–84: 0.42%<br>**Females**: 1,073 (91.01%)<br>**Work Settings** (top 4): PP/Contractual: 37.38%; Schools: 24.80%; Hospice: 19.43%; Psychiatry: 19.00%
Target client group	Top 4 populations of clients that respondents work with: Autism Spectrum Disorder: 44.73%; Developmental disabilities: 44.02%; Older Adults: 35.85%; Alzheimer's: 34.35%
Main outcomes (in relation to the scoping review)	**An open response question: Is there anything else that you would like to tell us about your experience since the COVID-19 outbreak?**<br>The most frequently mentioned topics within open comments included **virtual service delivery** (25.49%). Respondents discussing virtual services spoke more frequently about the benefits than the difficulties of such services, with many mentioning the lack of access to virtual service delivery options at the time of survey and some expressing mixed perspectives regarding this type of service delivery.<br>Regarding virtual services (the most frequent topic), some MTs expressed gratitude for the opportunity to continue earning money and providing services during this time of social distancing. Others expressed frustration that virtual service technology is difficult to learn, not appropriate for all clients, and cost prohibitive for many clients and clinicians. Additionally, MTs mentioned barriers and inequities, which kept clients from accessing these services.<br>MTs in private practice were impacted more than MTs in other settings.<br>**see table below**
Additional outcomes	Experiences, comments on **telehealth** (however, these are only examples, the study did not provide a full list of responses)<br>"I've been impressed by the quick pivot to telehealth by the field. While this isn't ideal and doesn't replace face to face MT, it is providing service to the clients."<br>"Standards have lowered even more in pretending that telehealth is effective."<br>"MT doesn't translate well online so I feel very limited."<br>**Changes in employment**<br>When providing information about changes in employment (n = 1,096), 210 (19.16%) respondents reported no changes to their work duties. Respondents reporting changes in employment (n = 886) were most often providing **remote clinical services** from home (67.40%). Fewer respondents reported being assigned different duties to complete on-site (14.78%) or from home (14.11%). One hundred and forty (15.83%) reported having their positions furloughed, and 23 (2.60%) reported having their positions eliminated.<br>**Change in contact hours**<br>Of the respondents who reported working at the time of this survey, 924 provided information about their hours at this time in the pandemic (compared with 1,135 before the pandemic). Sixty-one respondents (6.60%) reported having ≥30 client contact hours per week, compared with 244 (21.49%) prior to the pandemic. Eighty-six (9.31%) reported 10 to 29 client contact hours per week, in comparison to 373 (32.86%) prior to the pandemic. The greatest increase was seen in the category of 1 to 9 hours per week, with 395 (42.75%) compared with 199 (17.53%) before the pandemic. Figure 1 shows the change in contact hours by selected settings.<br>**Changes in services**<br>*Prior vs. Pandemic*<br>Individual services: 55.49% vs. 61.58%<br>Family services: approximately same according to graph<br>Group services: 38.93% vs. 24.97%

	Other services during the pandemic: 9.39% **(alternative services, such as prerecorded videos/material creation, staff support, and administrative tasks)**<br>**Alternative services**<br>Telehealth services (54.81%), virtual music lessons (17.01%), prerecorded songs/playlists (16.98%), and prerecorded video sessions (16.00%).<br>„Telehealth has, by necessity, become a way we can access people who are either in remote locations or who are among the very sick or immunocompromised."
Music therapy techniques	N/A
Examples of work with concrete clients in the text	N/A
Any recommendations for music therapists´ practice in COVID-19 pandemic	Additional information on telehealth delivery would be beneficial to inform the profession on how technology was used in service delivery, including platforms, difficulties, and changes to services.
Any other recommendations	
Conclusions of the study	In the face of increased collective stress and uncertainty due to the pandemic, the research team found it encouraging to witness evidence of support and resilience within the MT community.<br>Overall, this survey indicated that the pandemic has impacted many MTs. As such, it is the central hope from this research team that all MTs will gain the resources needed to endure this unparalleled season.

**Theme 1**	**Virtual Services**	**130**	**25.49%**	
	Focus on benefits	44	33.85%	"I'm surprised to discover how much I can do via ZOOM. It's forced me to be creative in ways I hadn't been before."
	Not available	27	20.77%	"The company I work for will not accept Telehealth."
	Focus of difficulties	23	17.69%	"Telehealth and learning how to handle technology has been an uphill battle and constant struggle."
	Mixed perspective	16	12.31%	"Telehealth is a great solution; however, not every client is appropriate for it."

Author, month+year, country	Knott, September 2020, USA
**Title**	**Virtual Music Therapy: Developing New Approaches to Service Delivery**
Type of reference and language	Journal article, English
Setting and services	Guidelines for developing new approaches to delivery of music therapy sessions during the COVID 19 pandemic.
Target client group	Clients of music therapy in healthcare systems, educational settings and communities.
Main objectives	This article describes the coauthors' threetiered scaffold model intended to support the program development and deployment of virtual music therapy (VMT) services.
Any additional objectives	Description of specific interventions utilizing a VMT approach during the pandemic.
Methodology	Not reported
Participants/Population (definition, age, gender, profession, size and characteristics of the sample(s)	N/A
Main outcomes	**THREE-TIERED SCAFFOLD MODEL = 1.Curate Online Resources, 2.Create Original Content, and 3.Implement Telehealth**<br>Tier 1: Curate Online Resources = Tier 1 identifying preexisting content (audio, videos, and music-making instructions) readily available online that reinforce the therapeutic uses of music or social and emotional learning concepts - Care should be taken to ensure that identified resources are appropriate, accessible, and effective in addressing patient/client goals.<br>• organizational considerations: understand the capability of a patient or client to access internet-based materials, as well as their relevance in addressing treatment goals. Music therapists may create word documents or PDFs directed to patients and families, which outline online resource recommendations. Communicating these documents electronically with source material hyperlinked may reduce barriers to content. If working within an organization, communication with those responsible for maintaining the website will benefit from a music therapist's expertise in identifying the most useful resources for the populations served.<br>Tier 2: Create Original Content = MT must create content that is suitable for tele practice (create new content, etc). Think of how their patients will react to content when presented online.<br>Organizational considerations: Music therapists must consider how to deliver original videos and audio to patients and clients. While compressed audio files may be emailed, videos will require either cloud-based delivery or hosting on a website, such as YouTube or Vimeo. Music therapists working in organizations should seek partnerships with colleagues working in marketing, social media, and digital health departments so that original content can be developed within guidelines that fit organizational objectives. By working collaboratively with others, music therapists can leverage an organization's preexisting social media networks to disseminate audio and video recordings for implementation by patients and clients.<br>Tier 3: Implement Telehealth = music therapists must consider which options best match the technical and physical capabilities of their clients and requirements of their payor. These platforms may include Zoom, Skype, Facetime, Doxy.me, and Microsoft Teams etc. MT also must ensure that clients have suitable technology for telehealth (laptop or computer, video camera, microphone, additionally headphones, USB stick etc.)<br>Organizational considerations: chief organizational considerations define what is allowable and required for billing. Private MTs should consult their state and payor to clarify telehealth guidelines. It is also recommended that music therapists consider utilizing private and password-protected platforms (nonpublic such as Zoom or Webex) in order to provide adequate security.<br>Operational considerations: assess their client's access and ability to use the required technology. consider caregiver or family member's availability to assist. the therapist will be required to identify their needs prior to the telehealth sessions: a computer or laptop, followed by additional considerations for Universal Serial Bus (USB) webcams, microphones, and audio interfaces. It is recommended that USB webcams have a minimum resolution of HD1080p. It is recommended that therapists utilize a USB-powered condenser microphone.<br><br>MT also should help client to find a suitable space from which client will access the virtual music therapy (ideally from a quiet room, good light etc.)
Additional outcomes	
Music therapeutic techniques	Finger play songs can be used to reinforce infants' and toddlers' motor and language development. Preschool and early childhood music can be used to teach pre-academic and academic concepts.

	Relaxation-oriented audio and video recordings may be created to support teens and adults in developing new coping skills.<br>Instrument-making and musical instrument learning demonstrations could be used to practice recreational skills.
Examples of work with concrete clients in the text	Child VMT for therapeutic play. A developmental music play session was conducted through video conferencing with<br>a hospitalized toddler receiving cardiac care with the objective of increasing arm use, specifically reaching. The screen sharing function was utilized to facilitate the child reaching to touch the screen when prompted in the song. After the parent reported the child had touched the screen, the music therapist would continue the song, providing cause and effect reinforcement and sustaining engagement in the treatment.<br>Adult VMT for anxiety. Utilizing an adult patient's bedside phone, a music-assisted relaxation intervention was facilitated in order to reduce anxiety. The patient was led through a scripted relaxation while the therapist spoke and facilitated instrumental acoustic guitar music in the background. Throughout the session, the patient was introduced to guided imagery, breathing techniques, and mindfulness-based strategies. Additionally, the patient was provided resources for free online-guided imagery tracks along with education on regimen and usage with examples provided including preparing for anxiety-inducing events, such as medical procedures, discharge, or times of day when the patient desires deepened relaxation such as at bedtime. The patient was noted to self rate pre-session anxiety at 8/10 and post at 6/10, revealing a clinically significant reduction of 2 points<br>VMT and behavioral health. A hospitalized adolescent with COVID-19 on a behavioral health unit was engaged in a video teleconference session to reinforce coping skills using therapeutic music instruction. During the session, the patient complained of difficulty getting a deep breath (dyspnea). The music therapist engaged the patient in therapeutic singing, which resulted in the patient reporting improved ease of breathing at the end of the session.
Any recommendations for music therapists´ practice in COVID-19 pandemic	• consider how to deliver original videos and audio to patients and clients<br>• consult state and payor to clarify telehealth guidelines.<br>• consider utilizing private and password-protected platforms (nonpublic such as Zoom or Webex)<br>• pursue relevant resources to inform regulatory, copyright, music licensing, and technical concerns<br>• assess client's access and ability to use the required technology<br>• consider caregiver or family member's availability to assist in the implementation of VMT<br>• From the therapist's equipment and technology standpoint, the therapist will be required to identify their needs prior to the telehealth sessions. Standard recommendations will begin with a computer or laptop, followed by additional considerations for Universal Serial Bus (USB) webcams, microphones, and audio interfaces. It is recommended that USB webcams have a minimum resolution of HD1080p. It is recommended that therapists utilize a USB-powered condenser microphone. It is recommended that therapists review equipment ratings thoroughly through online stores' customer reviews and critically compare the provided content when choosing audio equipment for purchase in order to avoid overspending. It is not recommended that monetary investments be made into additional professional recording software such as Garageband (mac) or Cubase (pc)<br>• Test software and check that the voice and instruments are clearly heard throughout the signal chain<br>• considering audio and visual space within which they are holding sessions (ample lighting, avoid silhouetting, avoiding visual disturbance such as clutter, free of other sounds, free of pets, etc.)<br>• Develop a procedure for contacting clients and a tip sheet for them to easily connect for the session.
Any other recommendations	HIPAA-compliant methods of service delivery during the state of emergency; however, always consult with your specific site or organization.
Conclusions of the study	The severity and lasting nature of this worldwide health crisis and its disruption of traditional service delivery models require clinicians and researchers to develop the most effective uses of VMT while considering its limits with regard to clinical populations and need areas. Adopting an ethical and safety-conscious stance enables music therapists to develop VMT services to best meet patient/client needs during the COVID-19 pandemic and beyond.

Table S25 Data extraction tool:.

**Author, month+year, country**	**Negrete, July - August 2020, USA**
**Title**	**Meeting the Challenges of the COVID-19 Pandemic: Virtual Developmental Music Therapy Class for Infants in the Neonatal Intensive Care Unit**
Type of reference and language	Journal entry, English
Setting and services	Hospital environment. Developmental music therapy class for Neonatal intensive care unit.
Target client group	Infants at Intensive Care Nursery and their parents/relatives
Main objectives	Describe the adaptation of developmental music class at the NICU to a virtual environment during the COVID19 pandemic.
Any additional objectives	
Methodology	Case report (NR)
Participants/Population (definition, age, gender, profession, size and characteristics of the sample(s)	N/A.
Main outcomes	Music therapist facilitates the virtual class from home and does not need to wear a mask.<br>The music therapy program has offered a unique opportunity for babies to see a face and mouth without a mask.<br>Another benefit is that more infants are able to attend at the same time, because they can participate from their rooms. For example, more infants with tracheostomies have attended the class because nurses do not have to coordinate respiratory support and transportation for the infants. In addition, infants who are on isolation precautions are able to participate; this would not be possible for an in-person class.<br>The online class also minimizes the amount of staff time required to help with the class because all the family or nurse needs to do is to log on.
Additional outcomes	
Music therapeutic techniques	Videoconference via Zoom, using a gallery view - for the infant, parents, other family members.<br>Techniques used have not changed:<br>Songs that introduced socialization.<br>Used singing and shakers.<br>Songs taught baby sign language (eg. more, all, done, mama)<br>Before each song, the music therapist would educate the parents on how to participate in the song with their child,providing hand-over-hand support, pointing to the different body parts the song was cuing, explaining how the song supported their infant's development, and demonstrating how the song could be used when not in a group setting.
Examples of work with concrete clients in the text	My son has truly found joy in music through our Zoom classes. He is moving his hands, smiling, paying attention to when the music stops or changes, and even shaking his shaker.<br>Class is now held twice a week for 20 minutes over Zoom with clients over 4 months CGA.<br><br>Through Zoom, M. has been able to maintain one of his favorite therapies, which is music therapy class. He has found his loud voice with singing along with Brianna. He also recognizes the faces of other participants as well as<br>Brianna. Outside of the class, we sing the songs from class, which he enjoys.
Any recommendations for music therapists´ practice in COVID-19 pandemic	Consult with parents and NICU staff about the best day and time for virtual classes.<br>Involve family members off site in virtual programming to encourage bonding.<br>• Invite families to watch a session first, before attending with their child.<br>• Determine the most appropriate infants for virtual music therapy sessions.<br>• Identify at least one on-site staff member (e.g., nurse or child life specialist) who can help families troubleshoot technical difficulties.<br><br>Connect with interpreting services to translate instructions when appropriate.<br>Remind families that they cannot take pictures of the screen if other patients are present, due to HIPAA rules.
Any other recommendations	When using telehealth, use only for infants greater than 4 months corrected gestational age.<br>Remember to set time limits when screen time is being used; it should not exceed 25 minutes, and the screen should be turned off if the infant shows signs of over stimulation.
Conclusions of the study	Creative solutions have emerged due to the social distancing and perhaps some of them could be sustained once the pandemic is over.<br>There are benefits to having a blended schedule of in-person and virtual developmental music therapy classes. A blended schedule will enable parents who are onsite and infants who are able to leave their

	rooms to meet together to learn, socialize and share. It will also provide a similar opportunity for infants who are not able to leave their rooms, and family members who are unable to be present in person, to experience shared learning and social contact in a virtual class.
