# Supplementary material for: Adaptation of Music Therapists’ Practice to the Outset of the COVID-19 Pandemic—Going Virtual: A Scoping Review
Source: Int J Environ Res Public Health. 2021 May 12;18(10):5138. doi: 10.3390/ijerph18105138 (PMC8151825; doi:10.3390/ijerph18105138)
Supplement: Supplementary file 1 [file ijerph-18-05138-s001.zip › Supplementary S4 Data charting table from websites and Recommendations.pdf]

Table S26: Statements, advice, and recommendations extracted from websites of MT associations

	Statements, advice, and recommendations extracted from websites of MT associations
**Deciding to use remote therapy**	
	You have a duty to consider all the implications of your client accessing music therapy via a remote means: Explore the situation with the client or carer; Re-contract boundaries. Consider how a client can contact you; Conduct only if you feel competent, find it safe, and have consent; for each client the smooth transition from an in-person setting to an online one will require different approach.
	Consider: Individual and sociocultural equity related to access to telehealth/therapy; Consent for telehealth music therapy; Security (cyber) for the technology/platform selected; Confidentiality (awareness of threats to confidentiality at each remote site); Facility policies or guidance on use of telehealth/therapy; Payer specific information regarding telehealth/therapy reimbursement; Current state laws (state recognition requirements), executive orders, and agency guidance surrounding telehealth/therapy service delivery.
	Consultation with an attorney or other advisor who is knowledgeable of the details of your practice as well as your specific state regulations.
**Inform/prepare clients and carers prior to the session.**	
	Inform: Online sessions are not meant as a permanent alternative method of holding sessions; they are being offered as a contingency service to ensure that support can continue to be offered.
	Ensure client safety: All clients should have access to the agreed software, a secure internet connection, and a private room to ensure confidentiality - agree upon family members to respect client's privacy during the session, make sure they do not overhear the conversation. It is best always to use headphones during sessions, if necessary, clients can play music/white noise as a background so no one can overhear their conversation; For clients aged under 18 who can access remote sessions independently, support staff or family member should be available for check-in at the start and end of the session and contactable by phone should there be any issues during a session; For clients of any age who are not able to access remote sessions independently, support staff or family member must be able to facilitate the session as guided by the therapist.
	It may be helpful to pre-warn your client that music-making online and over the phone can feel more difficult than music-making in person, so they may feel they would prefer to talk for the whole session - if so, that is ok.
	Eye contact - for some, making eye contact via video may be more challenging than in-person - communicate this with the client and ensure he is comfortable during the call.
**Types of music therapy to offer (adapting the sessions)**	
	It should be possible to run a remote session similarly to an in-person session. If your client does not have instruments available to them, they may wish to consider singing or talking as part of their work, if they can. If your client has instruments, they may wish to bring them to where they are, and they may wish to consider singing.
	In some cases, it may not be possible or desirable to offer music therapy exactly as usual. You may wish to consider other musical resources in your toolkit that may feel appropriate or useful to offer as part of a remote session. Some examples include: Songwriting; Adding lyrics over instrumentals or loops (pre-composed or devised in-session); Receptive music therapy, including sharing playlists; Guided Imagery in Music techniques (if you are a qualified GIM practitioner)
	While it may be desirable to live stream music as part of open access service, it is not recommended to advertise this as music therapy.
**Platforms and Softwares.**	
	It is your duty to ensure that any software you are using complies with policies regarding the privacy of client data and, if relevant, your employer's internal policies. Encrypted programs: Skype, Zoom, WhatsApp, FaceTime, Signal, Microsoft Teams. Unencrypted programs: Google Hangout, Microsoft Classroom
	Using YouTube or any open access streaming service (via Facebook, Instagram, or any other social platform) is not recommended.

	Zoom: 1. Make sure your meeting is password protected. 2. Avoid sharing the meeting ID where possible. 3. Set the screen sharing option to “host only” before the meeting begins. 4.
	Always enable a waiting room in case your client arrives early for their session.
	Group sessions: Holding group sessions using Zoom is recommended (conditional – waiting for feedback from music therapists).
**Running the session**	
	Background: Take steps to ensure a professional background is presented: a quiet, neutral space with no disturbances. No people or pets should be in the background. In addition, for online sessions, there should be no personal pictures or other objects in the background that would give a “home-like” feel. Both therapist and client shall always use the same location for the call.
	Timing: Remote sessions should take place at the same time in-person music therapy would usually take place. Your client should also be somewhere quiet, where they are unlikely to be disturbed.
	BAMT: Your client (or their carer) should be responsible for contacting you at the time their session is due to start. It is this way round, so the client has the option of "making the journey" to their therapy as they usually would.<br>YAHAT: Contact the client via text once you are ready to do a session, agree on who calls who - usually should be the therapist if not wished otherwise by the client.
	Make sure that everything you are doing is visible on the screen - in case you are doing something off-screen, inform clients of what you are doing, so it doesn't make them feel uncertain.
	You, as the therapist should let your client know when it is time to finish your session. It is then your client's (or carer’s) responsibility to hang up and finish the session, again to allow them to "leave" therapy as they usually would.

Appendix 4. Data charting table – websites of music therapy associations

Source	Recommendations
**BAMT Guidance for Music Therapists during the COVID-19 outbreak (pdf), https://www.bamt.org/about-british-association-for-music-therapy/covid-19-useful-information/bamt-guidance.html**	*Remote sessions*<br>It should be possible to run a remote session similarly to an in-person session. If your client does not have instruments available to them, they may wish to consider singing or talking as part of their work, if they can. It may also be helpful to state clearly to clients and carers that what is being offered is a contingency service to ensure that support can continue to be offered, and that it is not meant as a permanent alternative method of holding sessions. The following should be considered to ensure client safety (with particular thanks to Chiltern Music Therapy for these points):<br>● All clients should have access to the agreed software, a secure internet connection, and a private room to ensure confidentiality.<br>● For clients aged under 18 who can access remote sessions independently, a support staff or family member should be available for a check-in at the start and end of the session and contactable by phone should there be any issues during a session.<br>● For clients of any age who are not able to access remote sessions independently, a support staff or family member must be able to facilitate the session as guided by the therapist.
	Types of music therapy to offer<br>In some cases, it may not be possible or desirable to offer music therapy exactly as usual. You may wish to consider other musical resources in your toolkit that may feel appropriate or useful to offer as part of a remote session. Some examples include:<br>● Songwriting<br>● Adding lyrics over instrumentals or loops (pre-composed or devised in-session)<br>● Receptive music therapy, including sharing playlists<br>● Guided Imagery in Music techniques (if you are a qualified GIM practitioner)
	It may be possible to run online or phone music therapy sessions using one of the following software programs:<br>ENCRYPTED: Skype, Zoom, WhatsApp, FaceTime, Signal, Microsoft Teams<br>UNENCRYPTED but still suitable if consent is given and understood: Google Hangout, Microsoft Classroom You must continue to be mindful of GDPR compliance, and it may be worth checking if your software company has a statement on GDPR. For further information, please refer to the ICO and NCSC guidance.<br>Client safety must be maintained as part of online and phone sessions. Therapists running online or phone sessions must take steps to ensure a professional background is presented: a quiet, neutral space with no disturbances while sessions are taking place. No people or pets should be in the background. In

	addition, for online sessions, there should be no personal pictures or other objects in the background that would give a "home-like" feel.<br>BAMT would recommend that remote sessions take place at the same time in-person music therapy would usually take place. Your client should also be somewhere quiet, where they are unlikely to be disturbed.<br>If your client has instruments they may wish to bring them to where they are, and they may wish to consider singing. It may be helpful to pre-warn your client that music making online and over the phone can feel more difficult than music making in person, so they may feel they would prefer to talk for the whole session - if so, that is ok.<br>1. Your client (or their carer) should be responsible for contacting their therapist at the time their session is due to start. It is this way round so the client has the option of "making the journey" to their therapy as they usually would.<br>2. The session will continue as usually as possible. It is up to you and your client whether you hold your phone to your ear or whether you use the speaker phone function.<br>3. You as therapist should let your client know when it is time to finish your session. It is then your client's (or carer's) responsibility to hang up and to finish the session, again to allow them to "leave" therapy as they usually would.
	Further advice on using Zoom<br>In response to recent reports, both anecdotally and in the media, of users experiencing compromised settings when using Zoom, we would recommend ensuring the Advanced Settings are set to the following options for all remote sessions:<br>1. Make sure your meeting is password protected.<br>2. Avoid sharing the meeting ID where possible.<br>3. Set the screen sharing option to "host only" before the meeting begins.<br>4. Always enable a waiting room in case your client arrives early for their session.<br>Using the paid version of Zoom has increased options for sharing and advance security.<br>*Precautionary advice*<br>BAMT would not recommend using YouTube or any open access streaming service for music therapy sessions. While it may be desirable to livestream music as part of an open access service, BAMT would not recommend advertising this as music therapy, as this may give a false impression to some online users. For further guidance, please refer to the HCPC's Standards of Conduct, Performance and Ethics, which can be found here .
	Remote Group Sessions<br>BAMT would recommend holding group sessions using Zoom. More information on this to follow - at the time of first publication, we're still waiting on feedback from music therapists.
	Postponing Sessions<br>If you find you are unable to continue sessions in any form, you will be facing many conversations with clients, families and carers during this anxiety-provoking time. Scheduling diarised time to talk with families will ensure you have enough time to think through with everyone affected what the recent changes mean for them, and to identify and signpost towards any immediate support needed.
**BAMT,<br>COVID-19 and HCPC Standards of Conduct, Performance and Ethics**	Online Sessions<br>Therapists must make sure that they have the appropriate skill level in order to facilitate sessions online. They must be competent in the technology prior to use. If a therapist does not deem themselves competent then they must complete any relevant continuing professional development prior to engaging in online therapy sessions. It is the therapist's duty to ensure that any software they are using complies with GDPR and if relevant their employer's internal policies.
	Remote Sessions<br>Remote music therapy sessions may not be appropriate for every client. The therapist has a duty to consider all the implications of their client accessing music therapy via a remote means. Before deciding on any therapy arrangement the situation should be explored with the client or carer, if appropriate, including any re-contracting of boundaries that is necessary. Therapists should consider how a client can contact them if they are working 'out of office'. Online sessions should only be conducted if the therapist feels competent in facilitating a remote session and has deemed it safe and secure for both parties, as well as consent having been given by the client.
	Online Sessions<br>Prior to any online session the therapist must give clients any information that they need regarding how sessions have changed in regard to COVID-19. This ensures that clients are able to make informed decisions surrounding consent for therapy to take place as well as ensuring that it is in their best interest. Online therapy must be facilitated through a platform that is safe and secure and that the therapist feels competent to operate. Direct therapy

	must not be facilitated through a platform that is open access (such as live streams via YouTube, Facebook, Instagram or any other social media platform). Any platform that is used for therapy must be GDPR compliant.
	Only in exceptional circumstances, such as the therapist going on long-term leave or leaving the service, should a therapist delegate responsibility for their clients to another therapist.
	Online Sessions<br>Where therapy sessions are being conducted online BAMT would recommend, where appropriate, that the therapist has a check-in and check-out with a carer, parent or professional, working with the client at the start and end of the session. This will give the opportunity for any information sharing that is needed. If sessions are normally recorded, and this is an area that has been previously contracted with the client, then where possible the therapist should continue to record the sessions. Where it is not the norm for sessions to be recorded, BAMT would recommend that the therapist use their professional judgement to determine the appropriateness and the impact that recording the sessions could have on the therapeutic relationship both currently, as well as when sessions return to being face-to-face. Where sessions are to be recorded the therapist must ensure that the appropriate consent has been sought.
**AMTA, Music Therapy Practice Considerations with COVID-19https://www.musictherapy.org/music_therapy_practice_considerations_with_covid-19/**	**Teleservices Considerations**<br>• Be cognizant of HIPAA, FERPA, and other regulations and maintain compliance with these guidelines. Be aware that these guidelines may continue to change in terms of what is allowable as we transition to different stages of the pandemic. Keep in mind that during a pandemic there may be periods where stricter lockdown is reinstated, depending on the region.<br>• Consider licensing and regulation factors when providing services via technology across state lines.<br>• Consider copyright issues, licensing, and/or other permissions related to use of music and images when providing teleservices (as well as in person services<br>• Consider discussing and including music-based instruction for safety protocols (like use of masks) with service users during the final telehealth sessions before returning to in-person interactions.
**AMTA Telehealth/Therapy Statement, https://www.musictherapy.org/music_therapy_in_telehealth/**	Considerations for telehealth music therapy services include:<br>• Individual and sociocultural equity related to access to telehealth/therapy (See references in Appendix A for related discussions)<br>• Consent for telehealth music therapy<br>• Security (cyber) for the technology/platform selected<br>• Confidentiality (awareness of threats to confidentiality at each remote site)<br>• Facility policies or guidance on use of telehealth/therapy<br>• Payer specific information regarding telehealth/therapy reimbursement<br>• Current state laws (state recognition requirements), executive orders, and agency guidance surrounding telehealth/therapy service delivery<br>• Consultation with an attorney or other advisor who is knowledgeable of the details of your practice as well as your specific state regulations<br>For special education settings, guidance from AMTA legal counsel indicates that if music therapy is listed as a related service on a student's Individualized Education Plan (IEP), access to virtual music therapy services should continue if the school district is offering virtual general education and special education services.
YAHAT (tips for MT's) – translated from Hebrew<br><br>http://www.yahat.org/download/files/%D7%98%D7%99%D7%A4%D7%95%D7%9C%20%D7%9E%D7%A7%D7%95%D7%95%D7%9F%20-%20%D7%90%D7%99%D7%AA%D7%9F%20%D7%92%D7%99%D7%9C%D7%95%D7%A8%20%D7%9E%D7%99%D7%9C%D7%A8%20(2).pdf	It is key for the therapist to ensure a smooth transition from in person setting to an online one. For each client, this will require different approach and treatment. During first call, therapist should ensure that client is comfortable with the platform that is being used as well as find out whether client has access to all things necessary for a good quality session (headphones, microphone, camera, quiet room, good internet connection). The client should also be introduced to a contract between the therapist and client - GDPR, session recordings, tec.<br>Create contract - GDPR, negotiate about recording sessions, use headphones so other family members of MT can't hear what is going on during therapy sessions<br>Video call - contact client via text once you are ready to do a session, agree on who calls who - usually should be the therapist if not wished otherwise by client<br>Client's setting - agree upon family members<br>to respect client's privacy during session, make sure they don't overhear conversation, it is best to always use headphones during sessions. If necessary, clients can play music/white noise as a background so no one can overhear their conversation.<br>Location - both therapist and client shall always use a same location for call, with a reasonable background that is not distracting.<br>Eye contact - for some, making eye contact via video may be more challenging than in person - communicate this with the client and ensure he is comfortable during the call. Also make sure that everything you are doing is visible on screen - in case you are doing something off screen, inform clients of what you are doing so it doesn't make them feel uncertain.
