# Supplementary material for: Adaptation of Music Therapists’ Practice to the Outset of the COVID-19 Pandemic—Going Virtual: A Scoping Review
Source: Int J Environ Res Public Health. 2021 May 12;18(10):5138. doi: 10.3390/ijerph18105138 (PMC8151825; doi:10.3390/ijerph18105138)
Supplement: Supplementary file 1 [file ijerph-18-05138-s001.zip › Supplementary S5 Tips for music therapy activities.pdf]

Supplementary E
Tips for music therapy activities, resources and webinars related to the pandemic

(This list was prepared in November 2020.)

Country/association/URL source	URL links to resources
**New Zealand, https://www.musictherapy.org.nz/covid-19/**	The following from OTNZ-WNA links to a series of interactive workshops and the toolkit available to the public is at www.otnz.co.nz<br>https://www.youtube.com/watch?v=mCqJclmha9g&feature=emb_title<br>https://www.youtube.com/watch?v=GZsdLXsH6Lg&feature=emb_logo<br>https://www.youtube.com/watch?v=xOC9sIOmqUI&feature=emb_logo
**New Zealand, https://www.nataliejack.com/telehealth**	Telehealth resources:<br>https://www.crowdcast.io/e/xdqeg53b/register<br>https://www.crowdcast.io/e/rifeg3eh<br>https://www.crowdcast.io/e/telehealthupdates<br>Webinar (and webinar notes) on sound quality for telehealth sessions<br>https://www.youtube.com/watch?v=oDFkQpzqKD8&fbclid=IwAR2zAF9C4zMMr5SwXgaNfNXqmNdq2lLhoLjH_l1_I2W1i3DAHYZ-323AANs<br>https://ab7ba85a-9086-4bcf-aa80-ad88ebeb5154.filesusr.com/ugd/b3dde0_77f4620416da43958d6dbce0a6d7c53e.pdf
**Blog**	Tips on telehealth<br>https://www.su.edu/blog/2020/03/music-therapy-shifts-online-with-focus-on-telehealth/
**UK, www.bamt.org**	Many resources<br>https://www.bamt.org/resources/covid-19-useful-information
**EMTC, www.emtc-eu.com**	Tips for remote sessions<br>https://www.emtc-eu.com/news/2020/5/26/music-connects-us-the-use-of-music-and-the-role-of-music-therapy-during-a-pandemic-crisis-situation
**Israel, https://www.yahat.org/**	General info, tips and encouragement:<br>https://www.yahat.org/news/news.aspx?newsId=91
**BAMT Guidance for Music Therapists during the COVID-19 outbreak (pdf)**	*Apps and other online resources (with particular thanks to Irene LoCoco and Liz Coombes)*<br>*Available in AppStore or GooglePlay*<br>KORG has made the iKaossilator free for a limited time<br>Moog has made the Minimoog Model D Synthesiser free for a limited time<br>BandLab<br>Fingertip Maestro<br>NodeBeat<br>SoundForest<br>Blocs Wave<br>Bloom (paid)
**Chiltern Music therapy**	Digital MT services:<br>https://www.chilternmusictherapy.co.uk/services/digital
**BAMT, many resources**	Conducting online sessions, manuals, tips:<br>https://www.bamt.org/resources/covid-19-useful-information/online-and-phone-therapy
**USA, linked from AMTA**	Coronavirus Coping Kit for Music Therapy BusinesOwners<br>https://my.soundscapingsource.com/coronavirus
**AMTA webinars**	A webinar with case reports<br>https://www.youtube.com/watch?v=PJ8vwKF-zSA&feature=youtu.be
**AMTA webinar**	Music Therapy & Telehealth - Sponsored by the AMTA COVID-19 Task Force<br>Learning Objectives:<br>- Learn how to choose the best telehealth platform for your practice<br>- Discuss differences and similarities for the music therapy process in telehealth vs. in person services<br>- Identify potential barriers for effective music therapy via telehealth and how to address them<br>- Identify ethical issues related to the delivery of music therapy via telehealth

https://www.youtube.com/watch?v=YwSjNjf4IR4
